# Mechanical instability and interfacial energy drive biofilm morphogenesis

**DOI:** 10.7554/eLife.43920

**Published:** 2019-03-08

**Authors:** Jing Yan, Chenyi Fei, Sheng Mao, Alexis Moreau, Ned S Wingreen, Andrej Košmrlj, Howard A Stone, Bonnie L Bassler

**Affiliations:** 1 Department of Mechanical and Aerospace EngineeringPrinceton UniversityPrincetonUnited States; 2 Department of Molecular BiologyPrinceton UniversityPrincetonUnited States; 3 The Howard Hughes Medical InstituteChevy ChaseUnited States; Massachusetts Institute of TechnologyUnited States; Weizmann Institute of ScienceIsrael

**Keywords:** biofilms, mechanobiology, *V. cholerae*, morphogenesis, biomaterial, development, Other

## Abstract

Surface-attached bacterial communities called biofilms display a diversity of morphologies. Although structural and regulatory components required for biofilm formation are known, it is not understood how these essential constituents promote biofilm surface morphology. Here, using *Vibrio cholerae* as our model system, we combine mechanical measurements, theory and simulation, quantitative image analyses, surface energy characterizations, and mutagenesis to show that mechanical instabilities, including wrinkling and delamination, underlie the morphogenesis program of growing biofilms. We also identify interfacial energy as a key driving force for mechanomorphogenesis because it dictates the generation of new and the annihilation of existing interfaces. Finally, we discover feedback between mechanomorphogenesis and biofilm expansion, which shapes the overall biofilm contour. The morphogenesis principles that we discover in bacterial biofilms, which rely on mechanical instabilities and interfacial energies, should be generally applicable to morphogenesis processes in tissues in higher organisms.

## eLife digest

Engineers have long studied how mechanical instabilities cause patterns to form in inanimate materials, and recently more attention has been given to how such forces affect biological systems. For example, stresses can build up within a tissue if one layer grows faster than an adjacent layer. The tissue can release this stress by wrinkling, folding or creasing.

Though ancient and single-celled, bacteria can also develop spectacular patterns when they exist in the lifestyle known as a biofilm: a community of cells adhered to a surface. But do mechanical instabilities drive the patterns seen in biofilms?

To investigate, Yan, Fei, Mao et al. grew biofilms of the bacterium called *Vibrio cholerae* – which causes the disease cholera – on solid, non-growing ‘substrates’. This work revealed that as the biofilms grow, their expansion is constrained by the substrate, and this situation generates mechanical stresses. To release the stresses, the biofilm initially folds to form wrinkles. Later, as the biofilm expands further, small parts of it detach from the substrate to form blisters. The same forces that keep water droplets spherical (known as interfacial forces) dictate how the blisters evolve, interact, and eventually shape the expanding biofilm. Using these principles, Yan et al. could engineer the biofilm into desired shapes.

Collectively, the results presented by Yan et al. connect the shape of the biofilm surface with its material properties, in particular its stiffness. Understanding this relationship could help researchers to develop new ways to remove harmful biofilms, such as those that cause disease or that damage underwater structures. The stiffness of biofilms is already known to affect how well bacteria can resist antibiotics. Future studies could look for new genes or compounds that change the material properties of a biofilm, thereby altering the biofilm surface.

## Introduction

Many of the stunning morphologies that distinguish living entities do not arise exclusively from gene expression programs, but rather from overarching contributions from mechanical forces (Heisenberg and Bellaïche, 2013; Thompson, 1992; Yamada and Cukierman, 2007). Such morphomechanical processes include the formation of ripple-shaped leaves (Liang and Mahadevan, 2009), tendrils and flowers (Gerbode et al., 2012; Liang and Mahadevan, 2011), as well as the dorsal closure and apical constriction-mediated epithelial folding processes that take place during *Drosophila* embryonic development (He et al., 2014; Solon et al., 2009). One key feature is common to many of these morphogenic transformations: two or more layers of biomaterials are attached to one another but each grows at a different rate (Wang and Zhao, 2015). Inevitably, such growth mismatches generate mechanical stresses, and corresponding shape instabilities, which depend on the mechanical and other material properties of the biological constituents, as well as their geometries. Some examples include villi formation during the development of the human gut and formation of gyri and sulci during cerebrum development (Shyer et al., 2013; Budday et al., 2015; Tallinen et al., 2016).

Though ancient in their evolutionary origin, bacterial cells can also display intricate developmental patterns, particularly when they exist in the community lifestyle known as biofilms (Hobley et al., 2015; Humphries et al., 2017; Persat et al., 2015). Biofilms are surface-associated bacterial communities that are embedded in a polymer matrix (O'Toole et al., 2000; Thongsomboon et al., 2018) and are a predominant growth mode for bacteria in nature (Hall-Stoodley et al., 2004; Humphries et al., 2017). Biofilms can be beneficial, for example in waste-water treatment (Nerenberg, 2016), but they also cause significant problems in health and industry (Costerton et al., 1999; Drescher et al., 2013) because they are resistant to physical perturbations and to antibiotics (Kovach et al., 2017; Meylan et al., 2018). Biofilms on surfaces undergo morphogenic transformations, beginning as smooth colonies and, over time, developing complex morphological features (Beyhan and Yildiz, 2007). Genes specifying matrix components that enable polysaccharide production, cell-surface adhesion, and cell–cell adhesion are required for the morphological transition (Hobley et al., 2015). However, the underlying mechanisms that dictate how these biofilm matrix components direct overall morphology are not well-understood. One model focuses on the differential spatial regulation of genes encoding matrix components as the key driver of biofilm morphogenesis (Okegbe et al., 2014). Another model suggests that localized cell death serves as an outlet for mechanical stresses and thus determines biofilm morphology (Asally et al., 2012). Most recently, theory has been put forward to suggest the possibility that global mechanical instabilities are involved in the development of biofilm morphology (Zhang et al., 2016; Zhang et al., 2017).

Here, by combining quantitative imaging, biomaterial characterization, mutant analyses, and mechanical theory, we show that the mismatch between the growing biofilm layer and the non-growing substrate causes mechanical instabilities that enable the biofilm to transition from a flat to a wrinkled film, and subsequently to a partially detached film containing delaminated blisters. The sequential instabilities that the film undergoes, coupled with the generation and annihilation of interfaces, drive the evolution of biofilm topography. Our results demonstrate that bacterial biofilms provide a uniquely tractable system for the quantitative investigation of mechanomorphogenesis.

## Results

### A mechanical instability model for biofilm morphogenesis

**Figure 1.**
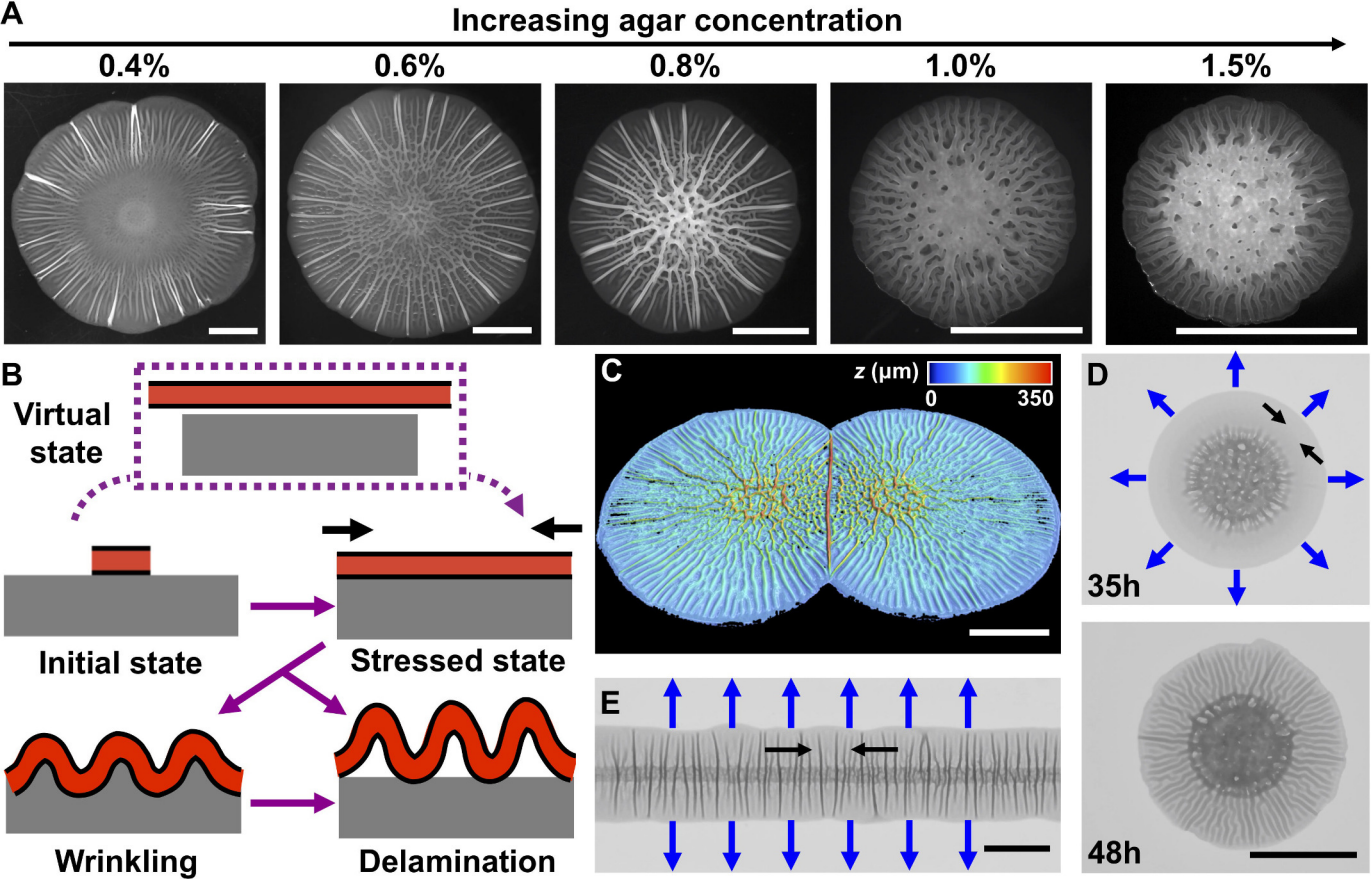
Mechanical instability drives *V. cholerae* biofilm morphogenesis. (**A**) Bright-field images of biofilms grown for 2 days on the designated percentages of agar. (**B**) Schematic of the wrinkling and delamination processes that occur during biofilm expansion. Red with a black outline denotes the biofilm. Gray denotes the substrate, agar in this case. (**C**) Three-dimensional (3D) profile of two colliding biofilms, initially inoculated 9 mm apart, grown on a 0.6% agar plate for 36 hr. (**D**) Transmission image of a *V. cholerae* biofilm grown for 35 hr (*top*) and 48 hr (*bottom*) on a 1.0% agar plate. (**E**) Transmission image of a *V. cholerae* biofilm inoculated as a line and grown for 30 hr on a 0.5% agar plate. In panels (**D**) and (**E**), blue arrows denote the expansion directions, and black arrows denote the tangential directions along which compressive stress accumulates. All scale bars are 5 mm.

Our central hypothesis is that biofilm morphogenesis is driven by mechanical instabilities that arise from the growth mismatch between an expanding biofilm and the non-growing substrate to which it adheres. To garner evidence for this idea, we grew biofilms on agar plates, which enabled us to control the mechanical properties of the substrate by changing the agar concentration (Nayar et al., 2012). We employed a commonly used *Vibrio cholerae* strain that lacks motility and constitutively produces biofilms (Beyhan and Yildiz, 2007; Yan et al., 2017). This strain (denoted WT in the present work) produces biofilms that have disordered cores decorated with radial features extending to the rims (Figure 1A). Indeed, biofilm surface morphology changes with increasing agar concentration: the spacing between the peripheral, radial features is reduced and their amplitudes become more homogeneous (Figure 1—figure supplement 1).

**Figure 1—figure supplement 1.**
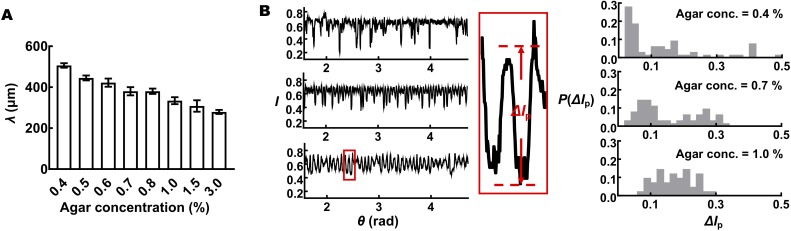
Quantification of *V. cholerae* biofilm surface morphologies. (**A**) The characteristic wavelength of the radial morphological features, *λ*, decreases with increasing agar concentration. (**B**) Transmitted light intensity profiles measured close to the outer edges of biofilms, *I*, show that the amplitudes of the radial features become more regular with increasing agar concentration. *Left*: typical profiles of transmitted light intensity, *I*, along the rims of 2-day-old biofilms for the designated agar concentrations. *Middle*: a closeup view of the extracted intensity profile from the bottom trace. The prominence of each peak, Δ*I*_p_, is used as a proxy for the height/amplitude of the corresponding radial feature. *Right*: probability distribution of Δ*I*_p_ for the designated agar concentrations. 10.7554/eLife.43920.005Figure 1—figure supplement 1—source data 1.Quantitation of *V. cholerae* biofilm surface morphologies.

**Figure 1—figure supplement 2.**
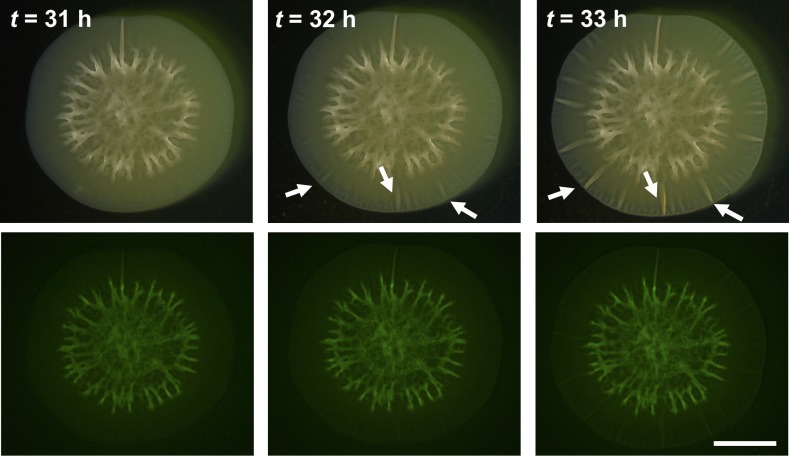
Wrinkling and delamination transitions are rapid. A biofilm grown on a 0.5% agar substrate was imaged at the designated times. *Top*: bright-field images. *Bottom*: fluorescent signal from the SytoX Green stain marking dead cells. White arrows indicate emerging morphological features. The wrinkling and delamination instabilities occur within a 2-hr window. No localized cell death was observed to precede the formation of wrinkles or blisters. Scale bar: 5 mm.

Encouraged by the observations described above and inspired by models developed to describe mechanical instabilities in abiotic materials systems (Li et al., 2012), here we propose a mechanomorphogenesis model for biofilms (Figure 1B). The biofilm originates as a flat film. Its volume increases over time due to cell proliferation and matrix production. If the biofilm were not attached to a substrate, it would grow into a stress-free state to cover a large area (Figure 1B, top, ‘virtual state’). However, the non-expanding agar substrate constrains biofilm expansion. Thus, biofilms are always subject to compressive stress (Figure 1B, middle right), which we hypothesize drives the surface morphology. Indeed, a biofilm growing at an air–liquid interface, not limited or compressed by a substrate, exhibits no surface features (Video 1).

**Video 1.** Part 1: A *V. cholerae* biofilm grown for 24 hr on 0.6% agar medium was peeled off of the substrate by the capillary method using LB medium as the liquid starting from the bottom left. The movie is played in real time. Part 2: The peeled biofilm from Part 1 grew at the air–liquid interface over time. Imaging began immediately after peeling and its total duration is 6 hr with 5-min time steps. The field of view is 73.0 mm × 48.3 mm.

According to mechanical instability theories, surface-adhered films under compression have several pathways to release compressive stress (Wang and Zhao, 2015). For example, the film can buckle out of the growth plane and deform together with the substrate into a periodically wrinkled pattern (Figure 1B, bottom left). In this mode, the compressive stress is released by film bending and substrate deformation. Alternatively, the film can directly delaminate from the substrate to form ‘blisters’ (Figure 1B, bottom right) (Vella et al., 2009), leaving the substrate essentially undeformed. An extra interfacial energy penalty is paid for delamination because new interfaces are generated, so direct delamination occurs in systems with film–substrate adhesion energies that are much smaller than their elastic deformation energies. Biofilms possess finite adhesion strength (~ 5 mJ/m^2^), which is the same order of magnitude as the deformation energy of the soft substrate (Yan et al., 2018). Thus, we suggest that biofilms could first wrinkle, and subsequently delaminate as growth gradually builds up compressive stress (Figure 1—figure supplement 2). According to this mechanomorphogenesis model, we should be able to change the biofilm topography by changing the spatial distribution of the mechanical stress. To this end, we inoculated two *V. cholerae* biofilms onto the same agar plate and allowed them to collide. Indeed, a large localized blister formed at the collision front where mechanical stress is most concentrated (Figure 1C; Video 2).

**Video 2.** Part 1: Collision of two *V. cholerae* biofilms grown on medium containing 0.6% agar. Imaging began 5 hr after inoculation and has a total duration of 75 hr with 15 min time steps. Biofilms were separated by 9 mm at the time of inoculation. At *t* = 20 hr, the biofilms begin to contact one another. The additional compressive stress present at the collision front leads to the formation of a large blister in the middle. The field of view is 41.5 mm × 27.7 mm. Part 2: Growth of a *V. cholerae* biofilm on medium containing 0.5% agar after cells were inoculated in a line. Imaging began 5 hr after inoculation and has a total duration of 72 hr with 15 min time steps. The field of view is 50.2 mm × 33.3 mm.

Our mechanomorphogenesis model provides an intuitive explanation for the commonly observed biofilm surface pattern of a disordered core surrounded by radial features at the edge (DePas et al., 2013; Okegbe et al., 2014; Wilking et al., 2013). Soon after the initial expansion of the biofilm, growth occurs primarily at the edge of the biofilm because of nutrient limitation at the center of the biofilm (Liu et al., 2015; Yan et al., 2017; and Figure 1—figure supplement 2). At the biofilm center, cell death has been shown to drive pattern formation (Asally et al., 2012). However, in the biofilm periphery, which is the region of focus of the current study, wrinkling and delamination occur with no preceding localized cell death (Figure 1—figure supplement 2). In this outer region, mechanical instabilities dominate the pattern formation and its wavelength. Directionality at the edge stems from the asymmetry between radial and tangential compressive stresses on the expanding front (Figure 1D). During cell proliferation, radial compressive stress is partially relieved by new biomass extending the biofilm boundary (Zhang et al., 2016). By contrast, in the tangential direction, compressive stress becomes concentrated because there is no analogous relaxation mechanism. Therefore, starting from a flat film, a growing biofilm will undergo mechanical instabilities predominantly in the tangential direction, leading to radial wrinkling, and later, to delamination patterns (Figure 1D). By contrast, in the interior region of a biofilm, compressive stress occurs in both the radial and tangential directions, giving rise to a network containing both radially and tangentially oriented features (Figure 1A,D). To demonstrate that pattern directionality is determined by expansion anisotropy, we changed the biofilm growth geometry by inoculating cells starting from a line so that the biofilm would extend quasi-unidirectionally (Video 2). In this geometry, compressive stress along the inoculation line is higher than that perpendicular to the line (the expanding direction). Therefore, wrinkles or blisters occur perpendicular to the biofilm line (Figure 1E).

### A trilayer mechanical model predicts the biofilm wrinkling wavelength

**Figure 2.**
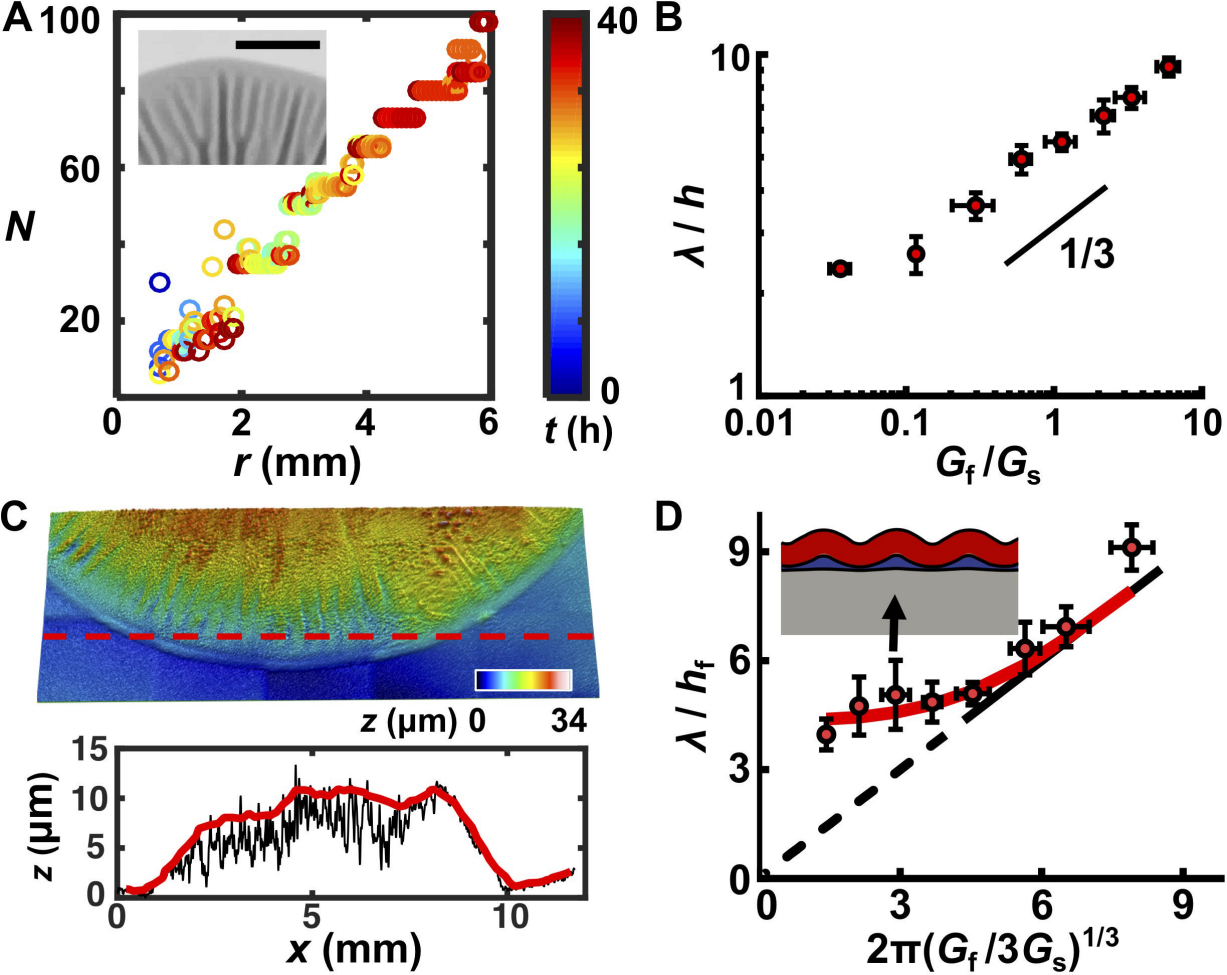
A trilayer mechanical model predicts the intrinsic wavelength of the biofilm pattern. (**A**) Number of wrinkles or blisters *N* versus the radial coordinate *r* during biofilm growth. The color scale indicates growth time *t*. Inset: closeup transmission image of a growing biofilm showing that wrinkles or blisters bifurcate to maintain a constant *λ*. Agar concentration: 0.7%, scale bar: 2 mm. (**B**) The scaling relationship between *λ* (normalized by the biofilm thickness *h*) and the shear modulus ratio *G*_f_/*G*_s_ between the biofilm and the agar substrate. The black line indicates a slope of 1/3 on a log-log scale. (**C**) Characterization of the residual layer. *Top*: 3D topography of the residual layer after peeling a biofilm off of an agar substrate. *Bottom*: height profile extracted along the contour indicated by the dashed red line in the *top* panel. Both the raw (black) and smoothed (red) data, from which the residual layer thickness *h*_r_ was calculated, are shown. Agar concentration: 0.5%. (**D**) Replot of the data in panel (**B**) taking into account the residual layer. The corrected biofilm thickness *h*_f_ was obtained by subtracting the residual thickness *h*_r_ from the total thickness *h*. The solid portion of the black line corresponds to the prediction from the bilayer model, which applies only to *x* coordinates greater than 4.75 (Wang and Zhao, 2015). The dashed portion of the black line is an extrapolation to zero from the bilayer prediction provided as a guide to the eye. The red line is the fitted data from the trilayer model in which the stiffness contrast between the residual and biofilm layers *G*_r_/*G*_f_ is treated as a fitting parameter while holding *h*_r_/*h*_f_ = 0.3. Inset: finite-element simulation of the trilayer model undergoing wrinkling instability. Red denotes the biofilm. Gray denotes the substrate. Blue denotes the residual layer. Simulation parameters were chosen to mimic the growth condition on 1.0% agar (black arrow). Data are represented as mean ± std with *n* = 3. 10.7554/eLife.43920.010Figure 2—source data 1.Experimental measuremants of biofilm residual layer thicknesses and wavelengths and predictions from trilayer wrinkling theory.

Mechanical instability theory predicts that, for a film–substrate system that is subject to compressive stress, the wrinkling wavelength is determined exclusively by the thickness and mechanical properties of the relevant materials (Huang et al., 2005). If so, we would expect the wrinkle wavelength to change with the mechanical properties of the biofilm and substrate but to be independent of the growth stage and geometry of the biofilm. To extract the wrinkle wavelength, we imaged the biofilm morphogenesis process over 72 hr and quantified the periodicity of radial stripes (Figure 2—figure supplement 1; Videos 3–5). We note that blisters emerge from wrinkles and that they inherit the wavelength of wrinkles, so we do not distinguish between the two in this analysis. We quantified the number of wrinkles or blisters *N* as a function of radial distance *r* from the biofilm center at different times. We found a linear relationship between *N* and *r* (Figure 2A, Figure 2—figure supplement 1). The slope has a geometrical origin: *N* = (2π/*λ*)*r* in which *λ* is the inherent wavelength of the system irrespective of the time in the developmental process or the location in the overall pattern (except at the biofilm core). A constant wavelength *λ* also means that radial wrinkles or blisters must bifurcate to maintain constant spacing as *r* increases, and indeed, we observed this to be the case (Figure 2A, inset). We also confirmed that the same *λ* was maintained when cells were inoculated in the line geometry and grew quasi-unidirectionally (Figure 2—figure supplement 1). We conclude that the wavelength of wrinkles or blisters reflects an intrinsic physical property of the biomechanical system.

**Video 3.** Growth of a *V. cholerae* biofilm on medium containing 0.4% agar. Imaging began 5 hr after inoculation and has a total duration of 75 hr with 15 min time steps. The field of view is 41.5 mm × 27.7 mm.

**Video 4.** Growth of a *V. cholerae* biofilm on medium containing 0.7% agar. Imaging began 5 hr after inoculation and has a total duration of 75 hr with 15 min time steps. The field of view is 41.5 mm × 27.7 mm.

**Video 5.** Growth of a *V. cholerae* biofilm on medium containing 1.0% agar. Imaging began 5 hr after inoculation and has a total duration of 72 hr with 15 min time steps. The field of view is 24.0 mm × 16.0 mm.

**Figure 2—figure supplement 1.**
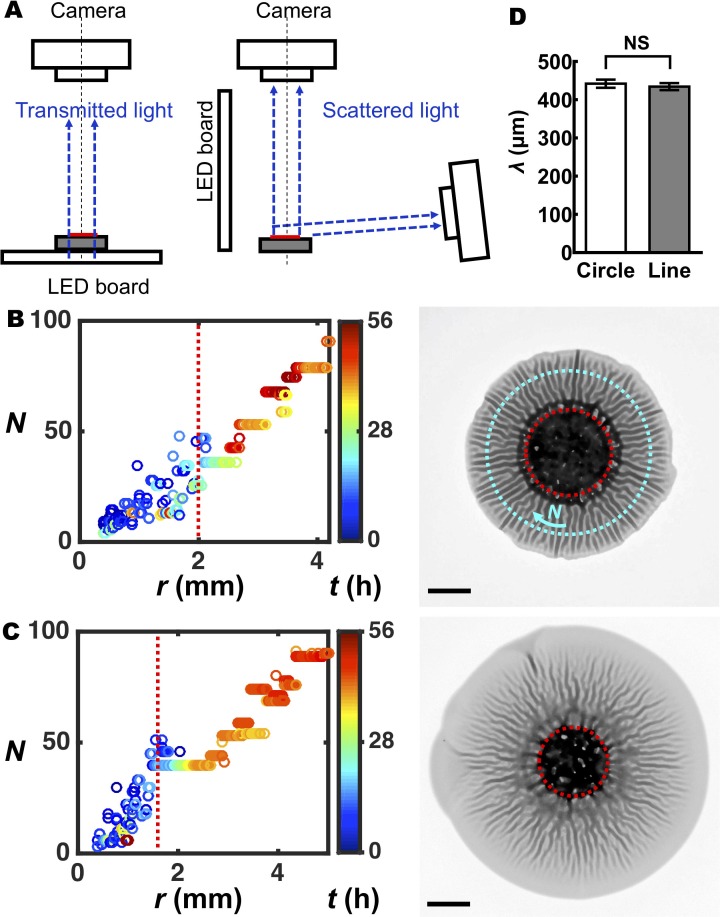
Analysis of intrinsic wavelengths in the morphologies of biofilms. (**A**) Experimental setups for imaging biofilm morphogenic development. *Left*: schematic of the imaging system used for the analysis of biofilm patterns and biofilm expansion. An agar plate with cells inoculated onto it was placed on a white-light LED board, under a camera that detects transmitted light. *Right:* schematic of the imaging system used for simultaneous acquisition of top and side views for analysis of biofilm morphogenesis. A growing biofilm was illuminated with a white-light LED board from the side, and two cameras were employed to detect the scattered light from above and from the side. In both setups, red denotes the biofilm and gray denotes the agar substrate. (**B,C**) Number of wrinkles or blisters *N* versus the radial coordinate *r* during biofilm expansion is plotted on the left, with the image of the corresponding biofilm at 48 hr shown on the right, for (**B**) agar concentration = 0.8% and (**C**) agar concentration = 1.0%. The color scale indicates growth time *t*. We note that the characteristic length of the pattern inside the nutrient-limited zones (outlined with the red dotted circles in panels (**B**) and (**C**), images on the right), differs from that of the morphological pattern outside of the zones because the morphology inside the biofilm core has been shown to arise from localized cell death (Asally et al., 2012; Yan et al., 2017). In panel (**B**), the cyan ring denotes a typical radius in the radial pattern region along which *N* was counted. Scale bars: 2 mm. (**D**) Comparison of the wavelengths of the patterns for biofilms grown on identical agar substrates (0.5% agar) but in different initial geometries (circle (white) versus line (gray)). Unpaired *t*-tests with Welch’s correction were performed for statistical analyses. p-value = 0.394. NS denotes not significant. Data are represented as mean ± std with *n* = 3. 10.7554/eLife.43920.012Figure 2—figure supplement 1—source data 1.Biofilm wavelength analysis.

**Figure 2—figure supplement 2.**
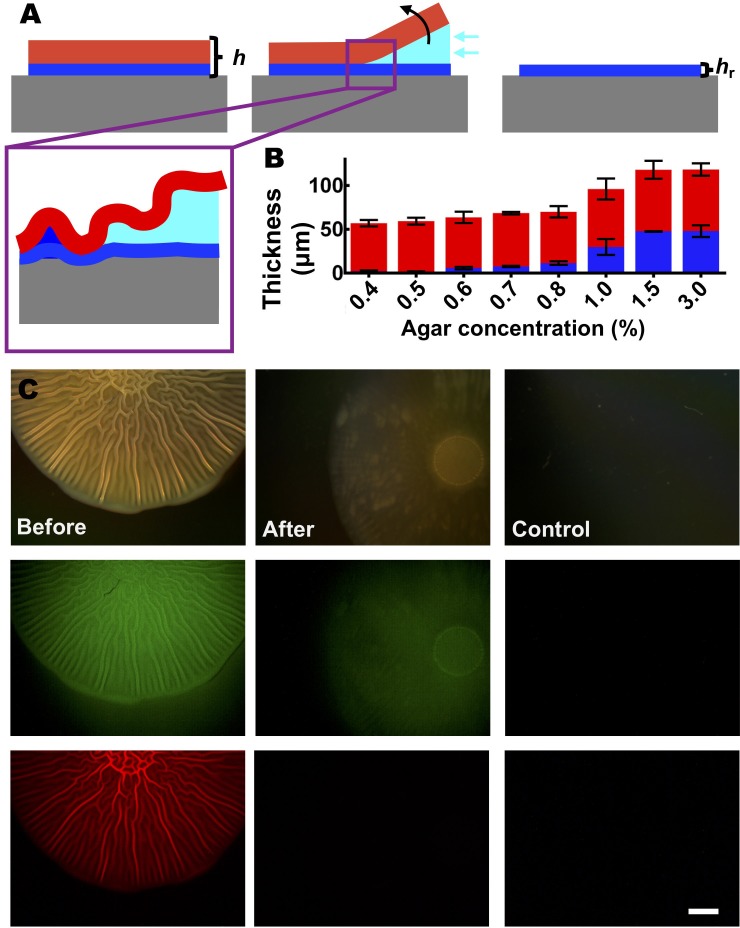
Capillary peeling reveals a residual layer between the biofilm and the substrate. (**A**) Schematic of the capillary peeling process (see also Yan et al., 2018). *Top*: the proposed trilayer biofilm model includes a residual layer (blue) between the biofilm layer (red) and the agar substrate (gray). Cyan represents the slowly injected liquid. The weak interface between the biofilm layer and the residual layer is separated by the penetrating liquid, leaving the residual layer on the agar substrate. *h* denotes the total thickness of the biofilm. *h*_r_ denotes the residual layer thickness. *Bottom left*: a closeup version of the schematic representation of liquid penetration between the biofilm and residual layers during the capillary peeling process. (**B**) Comparison of the corrected biofilm thickness *h*_f_ (red, obtained from *h*_f_ = *h – h*_r_) and the residual layer thickness *h*_r_ (blue), as a function of agar concentration. *h*_f_ changes minimally with changing agar concentrations. Thus, we hypothesize that the final thickness of the biofilm layer is set by the availability of oxygen, which can generally penetrate into a biofilm to a distance of tens of microns (Okegbe et al., 2014). Data are represented as mean ± std with *n* = 3. (**C**) The residual layer between the biofilm and the substrate contains primarily matrix material. *Top*: bright field images. *Middle*: Oregon green conjugated wheat germ agglutinin (WGA) staining for extracellular polysaccharide (Berk et al., 2012). *Bottom*: the signal from a constitutive *mKate2* transcriptional fusion that marks cells. A *V. cholerae* biofilm expressing *mKate2* was grown for 2 days on a 0.5% agar substrate, stained with WGA, and subsequently imaged (*left*). After the biofilm was peeled off the substrate using the capillary method from panel (**A**), the agar substrate was imaged again (*middle*). After biofilm peeling, a weak signal can be observed in the WGA channel but not in the *mKate2* channel, suggesting that the residual layer contains primarily extracellular polysaccharide but not live cells. As a control, an identically treated sterile agar substrate is shown (*right*). Scale bar: 2 mm. 10.7554/eLife.43920.014Figure 2—figure supplement 2—source data 2.Thicknesses of the biofilm and residual layers.

**Figure 2—figure supplement 3.**
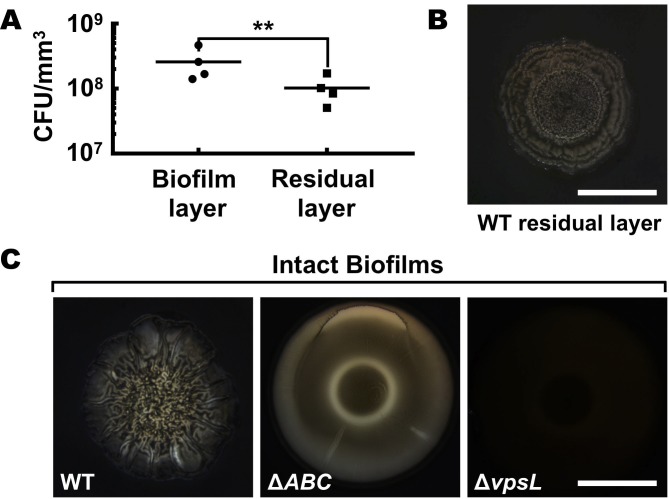
The biofilm residual layer consists primarily of polysaccharide. (**A**) Normalized CFU (colony forming units) in the biofilm and the residual layers for 2-day-old biofilms grown on a 0.6% agar substrate. Paired *t*-tests (*n* = 4) were performed for statistical analyses. ** denotes p-value < 0.01. (**B**) Bright field image of the residual biofilm layer from a WT biofilm counter-stained with India ink, which is excluded by polysaccharides. (**C**) Control experiments for panel (**B**). Bright field images of intact WT (*left*), Δ*rbmA* Δ*bap1* Δ*rbmC* (denoted Δ*ABC*, lacking key matrix proteins but possessing the key matrix polysaccharide) (*middle*) and Δ*vpsL* (lacking the key matrix polysaccharide) (*right*) biofilms counter-stained with India ink. India ink is excluded from the biofilm and residual layer when the key matrix polysaccharide is present (WT residual layer, WT biofilm, Δ*ABC* biofilm) but not when the key matrix polysaccharide is absent (Δ*vpsL* biofilm). The cells themselves do not exclude the India ink (as shown by the Δ*vpsL* mutant biofilm). We take these findings as preliminary evidence to suggest that the residual biofilm layer is primarily composed of polysaccharide. We do not exclude the possibility that the residual layer could also contain membrane, proteins, and other components. Scale bars: 5 mm. 10.7554/eLife.43920.016Figure 2—figure supplement 3—source data 3.Cell counts in biofilm and residual layers.

**Figure 2—figure supplement 4.**
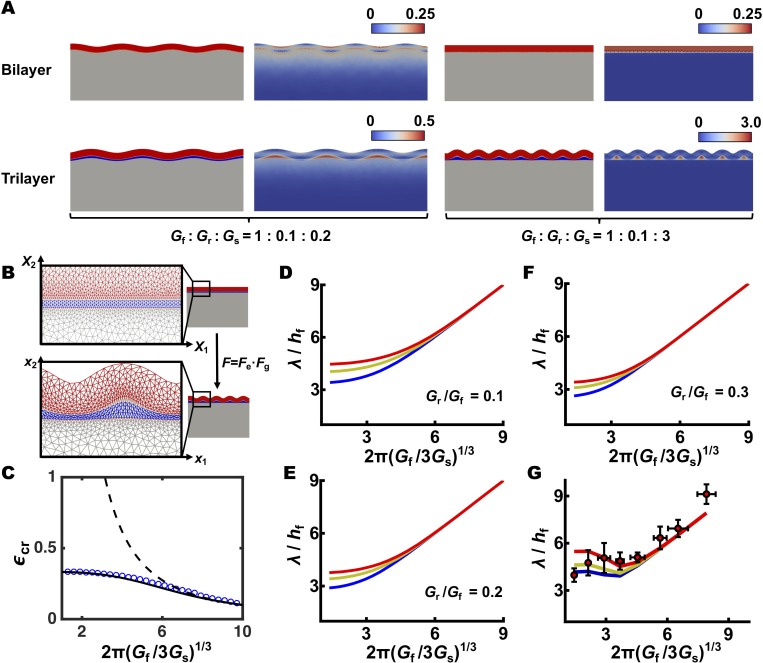
The trilayer biofilm morphology model predicts the wrinkling wavelength observed in the experiments. (**A**) Comparison of simulations of a bilayer model (*top*) and a trilayer model (*bottom*) following constrained biofilm growth. *Left*: both models predict surface wrinkling when the biofilm is stiffer than the substrate. *G*_f_/*G*_s_ = 5.0 and the growth-induced compressive strain *ε =* 0.2. *Right*: when the substrate is stiffer than the biofilm, the biofilm remains flat in the bilayer model, but wrinkles in the trilayer model. *G*_f_/*G*_s_ = 1/3 and *ε =* 0.32. Parameters for the residual layer in the trilayer model are: *G*_r_/*G*_f_ = 0.1 and *h*_r_/*h*_f_ = 0.3 in all simulations. For each simulation, the resulting configuration and strain distribution are shown side by side. First and third panels: color code as in Figure 2D. Second and fourth panels: color code denotes the von Mises equivalent strain (Jones, 2009). (**B**) Schematic of the trilayer simulation. Three layers are subdivided into finite elements (*left*). The coordinates in the initial and final deformed states are denoted by ***X*** and ***x***, respectively, and are connected by a deformation tensor ***F***. The total deformation can be further decomposed into two parts, one caused by growth ***F***_g_ and another by elastic deformation ***F***_e_. (**C**) Comparison of the critical compressive strain *ε*_cr_ for wrinkling predicted by bilayer and trilayer models. The bilayer wrinkling pattern would yield an *ε*_cr_ larger than one for small *G*_f_/*G*_s_ (corresponding to high agar concentrations in the biofilm growth experiment), which is physically inaccessible (dashed black curve). By contrast, the *ε*_cr_ predicted by the trilayer model (solid black curve, theory; blue circles, simulation) saturates at a value less than 1. Therefore, for small *G*_f_/*G*_s_ (<1.3), a wrinkling instability is predicted by the trilayer model but not by the bilayer model. (**D–F**) Theoretical predictions of the scaling relationships between the normalized wrinkling wavelength *λ*/*h*_f_ and the stiffness ratio *G*_f_/*G*_s_ in the trilayer model. The modulus ratio between the residual layer and the biofilm is constant (*G*_r_/*G*_f_ = 0.1, 0.2, and 0.3 for panels (D–F), respectively). The thickness ratio of the residual layer to the biofilm layer *h*_r_/*h*_f_ is varied from 0.1, 0.2, to 0.3, corresponding to the blue, yellow, and red curves, respectively. (**G**) As an alternative approach to the fitting procedure represented in Figure 2D, we used the experimentally determined *h*_r_/*h*_f_ and *G*_f_ for each growth condition and we varied the *G*_r_/*G*_f_ ratio from 0.1, to 0.2, to 0.3 as shown by the blue, yellow, and red curves, respectively. Experimental data (red circles) are represented as mean ± std with *n* = 3. 10.7554/eLife.43920.018Figure 2—figure supplement 4—source data 4.Theoretical and computational models for trilayer wrinkling.

Mechanical instability theory also predicts how the wavelength varies with the stiffness contrast between the biofilm and the substrate. Classical linear stability analysis for bilayer film–substrate systems predicts that *λ*, normalized by the film thickness *h*, should be equal to 2π(*G*_f_/3*G*_s_)^1/3^, in which *G*_f_ and *G*_s_ are the shear modulus of the film and the substrate, respectively (Chen and Hutchinson, 2004; Huang et al., 2005). The 1/3 power law is a result of the competition between the energy cost to deform the film and that to deform the substrate. To test whether this relationship applies to biofilms, we measured *λ*, *h*, *G*_s_, and *G*_f_ for all growth conditions. *G*_f_ varies minimally over a wide range of agar concentrations, whereas *G*_s_ varies by almost three orders of magnitude for agar concentrations from 0.4% to 3% (Supplementary file 1 Table S1). Plotting *λ*/*h* versus *G*_f_/*G*_s_ on a log-log scale (Figure 2B) reveals the characteristic scaling power law of 1/3, indicating the applicability of mechanical instability theory to biofilm morphogenesis.

One key discrepancy exists between the experimental measurements and the bilayer model. Bilayer theory predicts that, if *G*_f_/*G*_s_ < 1.3, the substrate is too stiff for the flat-to-wrinkling transition to occur (Wang and Zhao, 2015). However, wrinkling occurs in our experiments for *G*_f_/*G*_s_ well below 1.3, corresponding to agar concentrations ≥ 0.7%. To reconcile this discrepancy, we considered that a third soft, intermediate layer could exist between the growing biofilm and the non-growing substrate, which has been shown to allow wrinkling behavior even at low *G*_f_/*G*_s_ ratios (Lejeune et al., 2016a).

To acquire evidence for an intermediate layer, we employed a capillary peeling method in which biofilms are gently dipped into water and the capillary force peels the biofilm off the substrate without destroying the biofilm or the underlying surface (Figure 2—figure supplement 2) (Yan et al., 2018). Prior to peeling, using reflective confocal microscopy, the total biofilm thickness *h* was measured. After peeling, a residual layer remained on the substrate with a thickness *h*_r_ (Figure 2C). Our preliminary analysis suggests that this layer consists primarily of matrix polysaccharide (Figure 2—figure supplements 2 and 3). Thus, the corrected biofilm thickness *h*_f_ was obtained as *h*_f_ = *h – h*_r_. We replotted our data using *h*_f_ (Figure 2D, Figure 2—figure supplement 4). To rationalize the replotted curve, we took advantage of recent modeling efforts concerning multi-layer wrinkling phenomena (Lejeune et al., 2016a). The only unknown parameter in our work is the shear modulus of the residual layer, *G*_r_. In our theoretical model, we use a residual layer thickness *h*_r_ = 0.3*h*_f_, which was obtained from our experimental measurements, and we left *G*_r_/*G*_f_ as a fitting parameter (Figure 2—figure supplement 4). The trilayer model qualitatively and quantitatively captures our experimental observations. Qualitatively, with a soft intermediate layer, the wrinkling pattern persists even when the substrate becomes stiffer than the biofilm (*G*_s _> *G*_f_). Unlike the bilayer model, in which the substrate is deformed by the wrinkling film, in the trilayer model, the soft interfacial layer assumes the major share of the deformation, effectively reducing the substrate stiffness (Figure 2D, Figure 2—figure supplement 4) (Lejeune et al., 2016a). Quantitatively, predictions from the trilayer model recapitulate the prominent features of the revised plot: *λ*/*h*_f_ scales according to the bilayer model as 2π(*G*_f_/*G*_s_)^1/3^ for large *G*_f_/*G*_s_ ratios, but increasingly deviates from the 1/3 scaling law for smaller *G*_f_/*G*_s_ values. In the low *G*_f_/*G*_s_ regime, wrinkling is increasingly controlled by the soft intermediate layer. An intermediate layer stiffness of *G*_r_ = 0.1*G*_f_ allows the trilayer model to best fit our experimental data over all conditions.

### The biofilm wrinkling-to-delamination transition is controlled by interfacial energy and substrate stiffness

We next investigated the second transition predicted by our mechanomorphogenesis model: wrinkling-to-delamination. Whether and when a film–substrate system undergoes delamination is controlled by a competition between the adhesion energy between layers, Γ , and the elastic energy in the substrate. A dimensionless term Γ^*^, defined as Γ/(*h*_f_*G'*_s_) in which *G'*_s_ is the effective substrate modulus taking into account the residual layer (Lejeune et al., 2016a; see also Materials and methods), was used previously to quantify the relative importance of the two energies (Wang and Zhao, 2015). We recently measured the biofilm–agar interfacial adhesion energy Γ ~ 5–10 mJ/m^2^ (Yan et al., 2018). Hence, Γ^*^ is in the order of 0.01–1 in the current system, making delamination highly likely to occur during biofilm growth. In the context of the trilayer model, delamination takes place at the weakest interface, which is between the biofilm and the residual layer.

**Figure 3.**
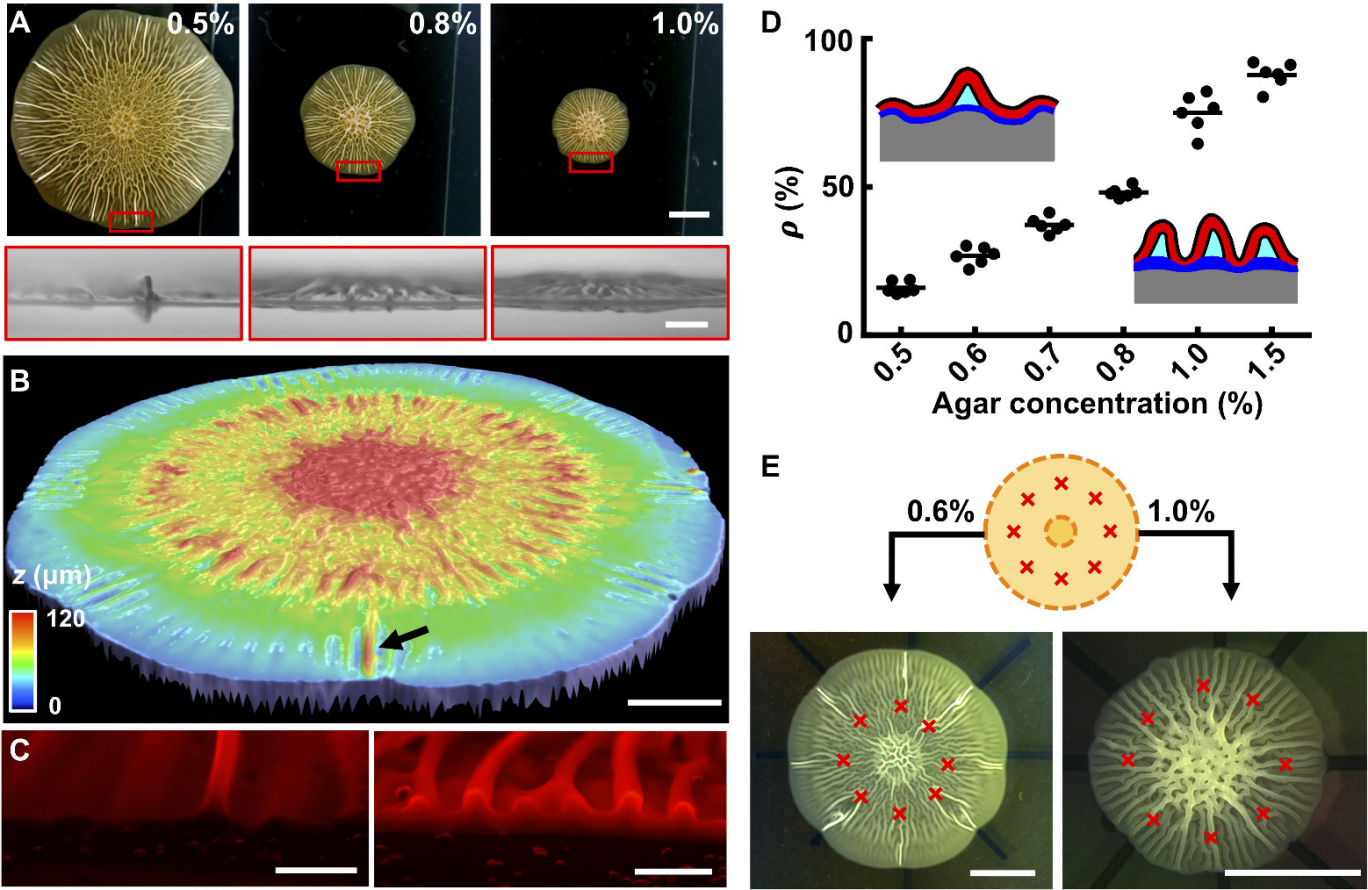
The biofilm wrinkling-to-delamination transition is controlled by adhesion energy. (**A**) Top (*top*) and side (*bottom*) views of biofilms on plates containing the designated concentrations of agar taken 10 hr after the onset of delamination. Scale bar: 5 mm (*top*) and 1 mm (*bottom*). (**B**) Surface topography of a biofilm grown on 0.5% agar at the onset of the wrinkling-to-delamination transition (36 hr). The arrow indicates a blister. Scale bar: 2 mm. (**C**) Cross-sectional views of rims of biofilms producing fluorescent mKate2, grown for 40 hr on plates containing 0.6% agar (*left*) and 1.0% agar (*right*). Scale bars: 0.5 mm. (**D**) Percentage (*ρ*) of blisters in all radially oriented features (wrinkles + blisters) versus agar substrate concentration for 2-day-old biofilms. The distinction between wrinkles and blisters is made on the basis of visual inspection. Insets: schematics showing how *ρ* depends on substrate stiffness. Red with black outline, biofilms; gray, agar substrate; blue, residual layer; cyan, liquid between the blisters and the agar. (**E**) Biofilm growth on a substrate with defined defects. *Top*: schematic. Yellow denotes the growing biofilm. Red crosses denote the eight defects that were generated by manually making holes in the agar. *Bottom*: bright-field images of typical experiments using the setup shown in the schematic (*top*), for biofilms grown on plates with the designated agar concentrations. Scale bars: 5 mm. 10.7554/eLife.43920.023Figure 3—source data 1.Wrinkles and blisters in biofilms.

To access the wrinkling-to-delamination transition experimentally, we simultaneously imaged the growing biofilm from the top and the side (Figure 3A, Figure 2—figure supplement 1). Radial wrinkles developed into blisters when growth proceeded beyond ~ 36 hr. At low agar concentrations, large amplitude blisters emerged among small amplitude wrinkles (Figure 3A). At higher agar concentrations, additional wrinkles developed into blisters, although with amplitudes smaller than those on low concentration agar substrates. We verified these findings using optical profiling to capture the full three-dimensional (3D) height information of the entire biofilm (Figure 3B). To peer inside blisters, we imaged cross-sectioned biofilms grown from cells producing fluorescence from *mKate2* (Figure 3C). At low agar concentration (i.e., 0.6%), only a small fraction of wrinkles were detached from the substrate in the form of blisters (Figure 3—figure supplement 1). By contrast, at high agar concentration (i.e. 1.0%), nearly all wrinkles had developed into blisters. In the cross-sectional images, voids were clearly present underneath the blisters, which were presumably filled with liquid (Wilking et al., 2013). Figure 3D quantifies the positive correlation between the percentage of wrinkles that converted to blisters at the biofilm edge and the substrate agar concentration.

**Figure 3—figure supplement 1.**
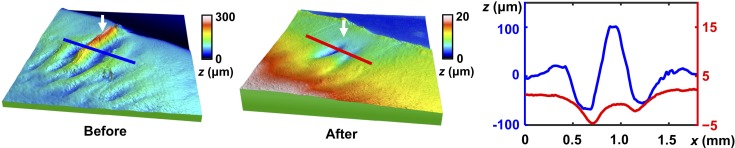
3D topography of a biofilm blister before and after capillary peeling. 3D height maps of a 4.60 × 3.50 mm region of a biofilm grown on 0.5% agar for 36 hr before (*left*) and after (*middle*) the biofilm layer was peeled off via the capillary peeling method. Red and blue lines indicate the positions from which the line profiles were extracted. White arrows indicate a blister.* Right*: line profiles at the indicated positions before (blue) and after (red) peeling. The zero position was chosen as the average height of the corresponding line profile. The capillary peeling process separates the weak interface between the biofilm layer and the residual layer, leaving the residual layer behind on the substrate. The location at which the blister was originally located becomes flat after biofilm removal. This observation suggests that there was no solid (either residual layer or agar substrate) filling the void under the blister, rather, the biofilm layer is locally delaminated from the surface underneath it. In addition, two large dimples are observed in the residual layer immediately adjacent to the position of the original blister, indicating that the biofilm had been significantly deformed into the residual layer. This finding is consistent with our observation that compressive strain is concentrated in the region containing a blister. Presumably, the large bending moment at the base of each dimple causes a large stress. 10.7554/eLife.43920.025Figure 3—figure supplement 1—source data 1.Height profile of a large blister before and after capillary peeling.

To rationalize the dependence of the delamination pattern on agar concentration, it is useful to recall the notion of normalized adhesion energy, Γ^*^. On stiff substrates, Γ^*^ is small, so delamination is favored over wrinkling. Blisters form extensively but they are small because they share the overall compression. On soft substrates, Γ^*^ is large, so blisters form only infrequently while the majority of the biofilm remains attached to the substrate. In this case, the isolated blisters concentrate the compressive strain and become larger than those on a stiff substrate. We hypothesized that the locations of blisters on soft substrates are defined by surface defects that trigger local delamination. This hypothesis is consistent with the observed heterogeneous sizes of blisters in biofilms grown on soft substrates. Specifically, we argue that blisters emerge at different times and at different locations in growing biofilms depending on when a surface defect is encountered during biofilm expansion. The different ages of blisters naturally lead to their heterogeneous heights. To test this possibility, we made surface imperfections in the soft agar substrate at defined positions (Figure 3E). Indeed, these imperfections dictated the exact locations at which blisters formed as the biofilm expanded. By contrast, on stiff substrates, delamination occurred along the entire biofilm rim, irrespective of the predefined surface imperfections (Figure 3E).

### Interfacial energy controls blister development dynamics and interactions between blisters

**Figure 4.**
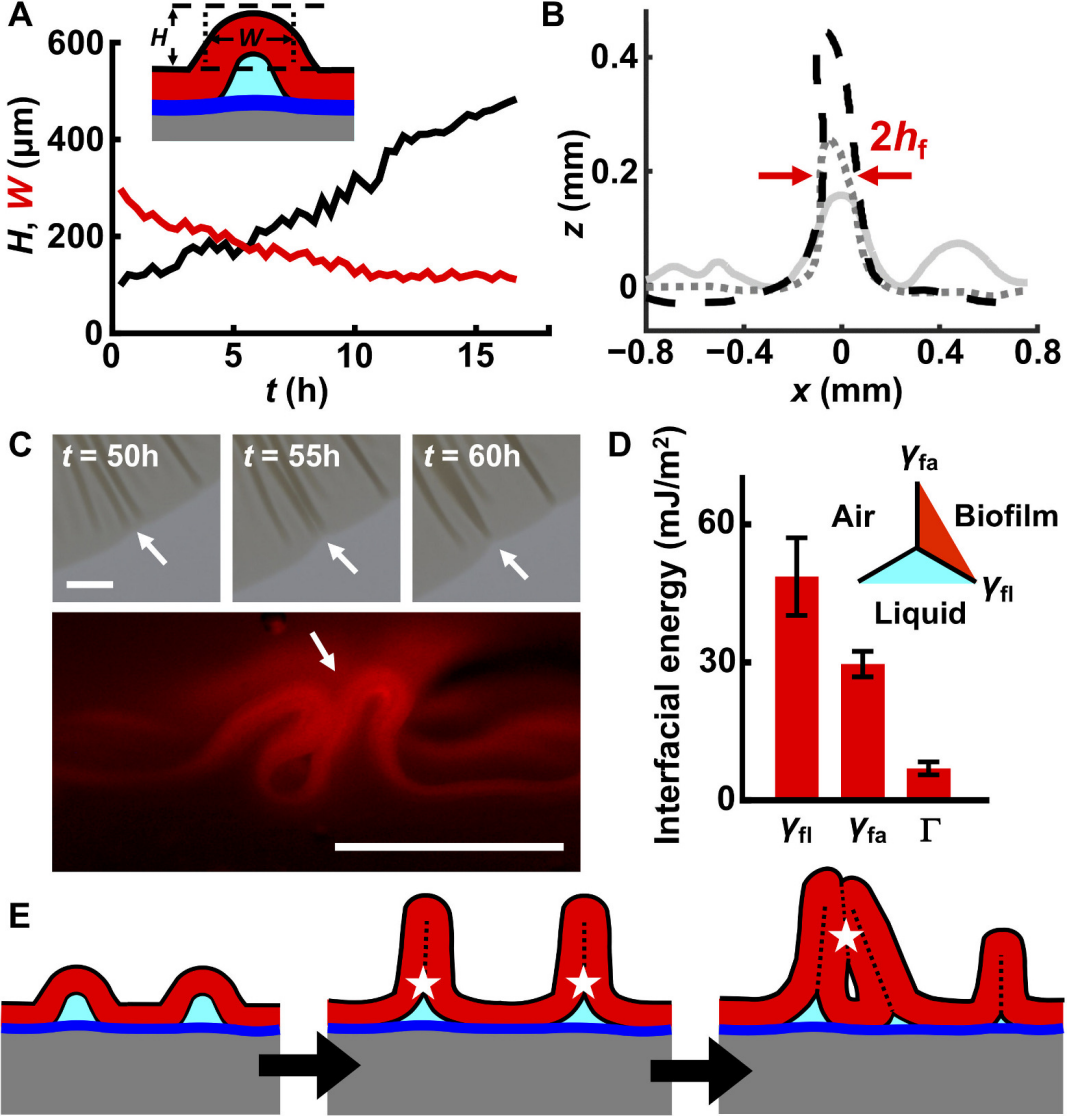
Interfacial energies control blister dynamics and interactions between blisters. (**A**) Time evolution of the height *H* (black) and width *W* (red) of a representative biofilm blister. Inset: schematic representation of a blister; color code as in Figure 3D. (**B**) Developing profile of a single blister, extracted from side view images at successive time points after delamination. Profiles are shown at 2.5 hr (gray line), 10 hr (gray dotted line) and 17.5 hr (black dashed line) after the onset of delamination. The distance between the red arrows corresponds to *W*, which, over time, approaches twice the biofilm thickness (2*h*_f_). Regions near the blister become flatter as cell mass is pulled into the blister. Agar concentration: 0.4%. (**C**) Representative merging of adjacent blisters (white arrows) at specified times (*top*). Cross-section image from a biofilm producing fluorescent mKate2 reveals blister peak-to-peak contact (*bottom*; designated by the white arrow). Agar concentration: 0.7%. Scale bars: 1 mm (*top*) and 0.5 mm (*bottom*). (**D**) Interfacial energy of the biofilm–air interface γ_fa_, biofilm–liquid interface γ_fl_, and the adhesion energy between the biofilm and the substrate Γ for WT *V. cholerae* biofilms. Data are represented as mean ± std with *n* = 3. *Inset*: schematic of different interfaces. (**E**) Schematic of blister development in a WT *V. cholerae* biofilm. White stars and dashed black lines denote interface annihilation events. For panels (**D**) and (**E**), the color code is the same as that in Figure 3D. 10.7554/eLife.43920.027Figure 4—source data 1.Blister formation and evolution dynamics and related interfacial energies in WT *V. cholerae* biofilms.

In conventional materials systems, a blister initially assumes a sinusoidal profile and then continues to grow in both width and height as the strain mismatch between the film and substrate increases (Vella et al., 2009). We wondered how blister width and height would develop in a living biofilm as the biofilm expands and accumulates strain mismatch. To examine this, we tracked isolated blisters by imaging the rim of the expanding biofilm (Figure 2—figure supplement 1). The width of each biofilm blister decreased while its height increased over time until the final width of the blister reached twice that of the thickness of the biofilm (Figure 4A,B). This final value for the blister width indicates that the two sides of the blister come into contact with one another. Subsequently, blisters continue to develop only in height. Moreover, large blisters suppress nearby wrinkles from delaminating (Figure 4B), presumably because the biofilm and the substrate can slide relative to one another such that a blister captures nearby compressed biofilm material, and in so doing, releases compressive stress in the vicinity. Neighboring blisters tend to merge during late stages of biofilm development (>48 hr), forming single dark features in the transmission images (Figure 4C (*top*) and Figure 4—figure supplement 1). Indeed, cross-sectional images reveal that head-to-head contact occurred (Figure 4C (*bottom*)).

**Figure 4—figure supplement 1.**
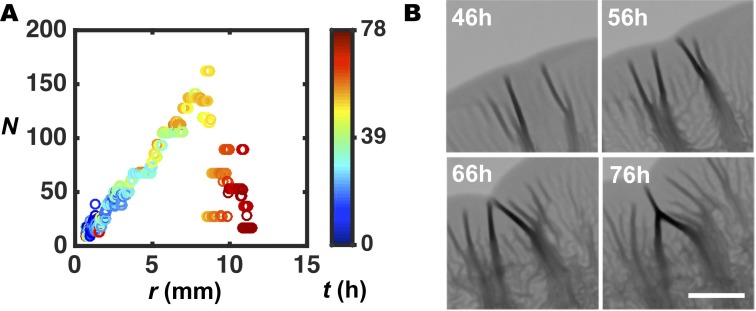
Characterization of pattern merging events. (**A**) Number of wrinkles or blisters *N *versus radial coordinate *r*, for the biofilm shown in Figure 2A over a longer time interval. *N* sharply declines at the rim during the late stages of biofilm growth because of the merger of adjacent blisters, which eliminates biofilm–air interfaces (Figure 4C). (**B**) Representative images of the rim of a WT *V. cholerae* biofilm grown on 0.4% agar at the designated times. Neighboring blisters are pushed toward each other by the adjacent flat regions. Ultimately, the blisters merge. Scale bar: 2 mm. 10.7554/eLife.43920.029Figure 4—figure supplement 1—source data 1.Wavelength analysis over three days of biofilm development.

**Figure 4—figure supplement 2.**
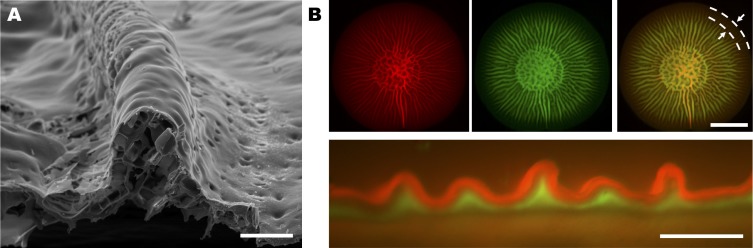
Analysis of the internal structures of biofilm blisters. (**A**) SEM image of a cross-section of an isolated blister from a 2-day-old biofilm grown on a 0.6% agar substrate. The two inner faces of the blister contact one another, and an empty space exists underneath the blister. Scale bar: 100 μm. (**B**) Images of a *V. cholerae* biofilm expressing *mKate2* grown for 2 days on a 0.8% agar substrate containing SytoX Green. The top view (*top*) and the cross-sectional view (*bottom*) are shown. *Top left*: signal from the constitutive *mKate2* transcriptional fusion that labels live cells. *Top middle*: signal from SytoX Green that stains dead cells. *Top right*: merged signals. Dashed lines and arrows in the *top right* panel indicate the annulus at the biofilm edge that contains a lower fraction of dead cells relative to the region internal to this annulus. The existence of such an annulus shows that cell growth occurs primarily at the edge of the biofilm. *Bottom*: cross-sectional view of biofilm blisters reveals stratification between live (red) and dead (green) cells. The sides of blisters contact one another via the dead cell layer. In panel (B), scale bars: 5 mm (*top*) and 500 μm (*bottom*).

**Figure 4—figure supplement 3.**
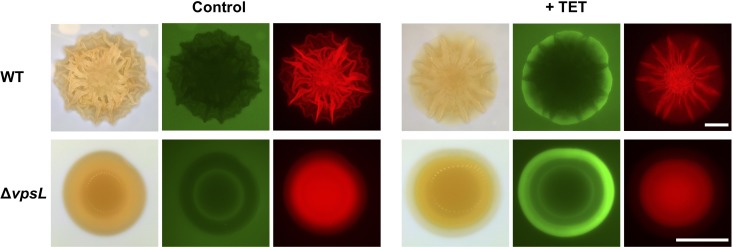
Bacterial cells residing in biofilm blisters are protected from antibiotics. Biofilms of cells constitutively expressing *mKate2* were grown on semipermeable membranes on top of a 0.6% agar substrate for 2 days. The membranes were transferred to the surface of LB liquid medium containing SytoX Green without (*left*) or with (*right*) 50 μg/mL tetracycline (TET) overnight. For each condition, the *left* part shows the bright-field images, the *middle* part shows SytoX staining of dead cells, and the *right* part shows the *mKate2* signal from live cells. In the absence of tetracycline, both WT and Δ*vpsL* mutant biofilms harbor few dead cells. In the presence of tetracycline, significant cell death occurs at the edges of both the WT and the Δ*vpsL* biofilms. However, WT cells in the biofilm regions containing blisters are less susceptible to the lethal effects of antibiotics than are cells in the intervening flat regions, presumably because cells residing in blisters are located further away from the antibiotic source than are cells that are not in blisters. Such variation in survival in the tangential direction does not occur in the Δ*vpsL* mutant biofilm, which possesses a smooth, blister-less morphology. Scale bars: 2 mm.

The sequential biofilm blister dynamics described above involve the generation or annihilation of new or existing interfaces, which have energy penalties or payoffs. To understand the order of these events, we measured their interfacial energies in WT *V. cholerae* biofilms (Yan et al., 2018). They are: biofilm blister–liquid underneath, *γ*_fl_ ~ 49 mJ/m^2^; biofilm blister–air above, *γ*_fa_ ~ 30 mJ/m^2^; and the energy needed to separate the biofilm from the residual layer underneath, Γ ~ 5 mJ/m^2^ (Figure 4D). This energy hierarchy determines the sequence through which interfaces are generated or annihilated (Figure 4E). First, compressive stress leads to delamination of the biofilm from the residual layer, forming a local blister. This step generates an additional high-energy interface between the blister and the liquid underneath it. To eliminate this high-energy interface, the two sides of the inner face of the blister come into contact with each other as the blister grows. Indeed, electron microscopy imaging of the cross-section of a blister shows this to be the case (Figure 4—figure supplement 2). After internal contact occurs, the blister can only develop in the vertical direction. However, blister growth enlarges the interface between the biofilm and the air. Subsequent merging of neighboring blisters (Figure 4C) eliminates biofilm–air interfaces, and in so doing, lowers the free energy of the entire system. An added benefit to the bacteria stems from these blister dynamics: cells in blisters are less susceptible to the lethal effects of antibiotics that diffuse in from the substrate than are cells residing in the base of the biofilm, presumably because cells in blisters are located further away from the antibiotic source (Figure 4—figure supplement 3).

**Figure 5.**
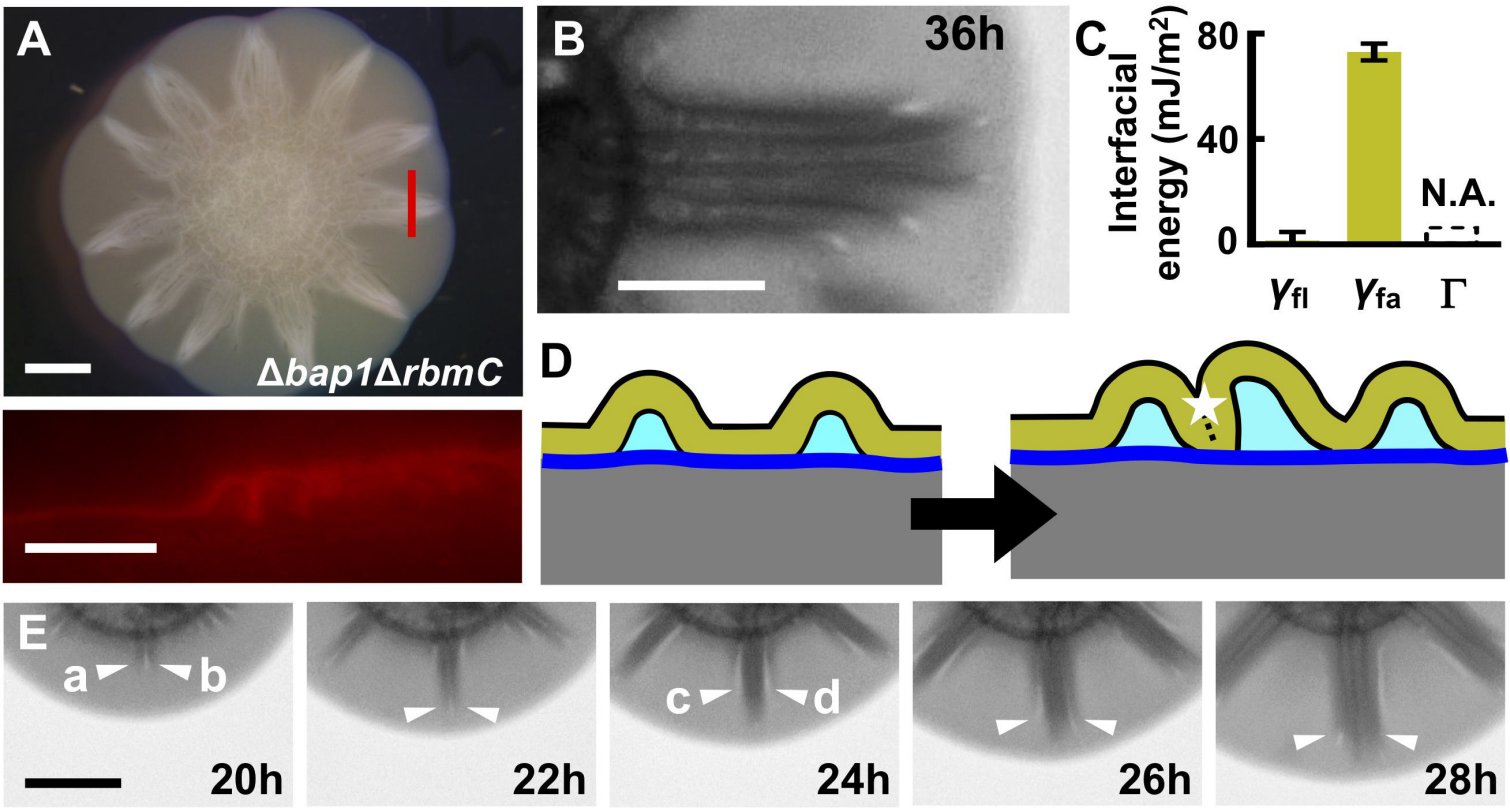
Morphogenesis of a mutant biofilm possessing altered interfacial energies. (**A**) Bright-field (*top*) and cross-sectional (*bottom*) images of a *V. cholerae* Δ*bap1*Δ*rbmC* mutant (abbreviated as Δ*BC* below) biofilm producing fluorescent mKate2, grown for 2 days on a 0.6% agar substrate. The red line in the *top* panel indicates the location of the cross-section used for the *bottom* panel. Scale bars: 2 mm (*top*) and 500 μm (*bottom*). (**B**) Close-up view of a star facet in a Δ*BC* biofilm grown on 0.6% agar for 36 hr. Scale bar: 1 mm. (**C**) Interfacial energies measured for the Δ*BC* biofilm. N.A. means too small to be measured. Data are represented as mean ± std with *n* = 3. (**D**) Schematic representations of Δ*BC* biofilm morphology development. Color code as in Figure 3D, except that yellow represents the Δ*BC* biofilm. (**E**) Transmission images of a section of a Δ*BC* biofilm growing on a 0.6% agar plate at the designated times. White arrowheads indicate emerging blisters. Four blisters (**a–d**) emerged during the time shown. Scale bar: 1 mm. 10.7554/eLife.43920.033Figure 5—source data 1.Interfacial energies of *V. cholerae* Δ*bap1*Δ*rbmC* mutant biofilms.

If the above interpretations concerning the involvement of interfacial energy in blister development are correct, changing the relative magnitudes of the three interfacial energies should modulate blister dynamics, and, in turn, the global biofilm morphogenesis process. To test this idea, we deleted *bap1* and *rbmC*, which encode proteins that are responsible for cell-surface interactions and biofilm hydrophobicity (Fong and Yildiz, 2007; Berk et al., 2012; Hollenbeck et al., 2014). Rather than forming isolated blisters, when formed on soft agar substrates, the Δ*bap1*Δ*rbmC* biofilm exhibits a star-shaped morphology with flat regions between the facets of the stars (Figure 5A (*top*)) (Yan et al., 2017). The cross-section of a single facet shows that it consists of a group of congregated blisters (Figure 5A (*bottom*)). Curiously, in contrast to the WT blisters, in the mutant, only the external surfaces of neighboring blisters are in contact with one another, leaving the internal spaces under each blister intact. Indeed, transmission images show that multiple stripes exist within one facet, corresponding to multiple blisters (Figure 5B, Figure 5—figure supplement 1).

**Figure 5—figure supplement 1.**
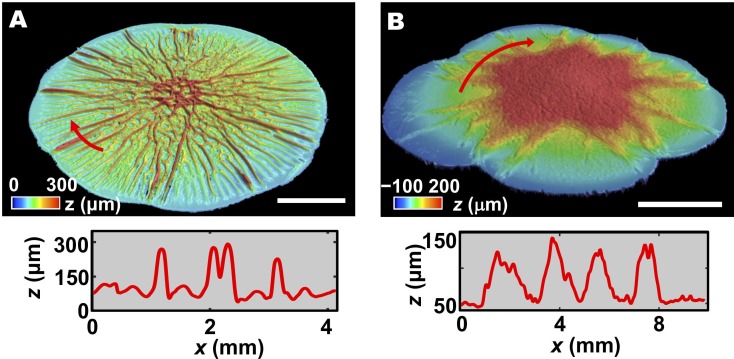
Interfacial energies determine the morphological features of the biofilm. (**A, B**) *Top*: 3D profile of a 2-day-old WT *V. cholerae* biofilm (**A**) and a 36-h-old Δ*BC* biofilm (**B**) grown on a 0.6% agar substrate. Scale bars: 5 mm. *Bottom*: height profiles extracted from the positions spanned by the red arrows in the *top* panels. In panel (**A**), several large blisters can be observed among the smaller amplitude wrinkles. In panel (**B**), several subpeaks are present within one large peak, indicating aggregation of blisters in the Δ*BC* biofilm. 10.7554/eLife.43920.035Figure 5—figure supplement 1—source data 1.Height profiles of WT *V. cholerae* and Δ*bap1*Δ*rbmC* mutant biofilms.

To rationalize the Δ*bap1*Δ*rbmC* blister dynamics, we measured the relevant interfacial energies (Figure 5C). The adhesion energy Γ between the Δ*bap1*Δ*rbmC* biofilm and the substrate is below the detection limit, meaning that delamination occurs more easily in the Δ*bap1*Δ*rbmC* biofilm than in the WT biofilm. Indeed, Δ*bap1*Δ*rbmC* biofilm blisters emerge directly from the expanding flat film, skipping the wrinkling state (Video 6). Second, the relative order of interfacial energies changes in the mutant: *γ*_fl_ approaches zero whereas *γ*_fa_ is large, consistent with the hydrophilicity of the Δ*bap1*Δ*rbmC* biofilm (Hollenbeck et al., 2014). These alterations in interfacial energies have profound consequences for blister dynamics (Figure 5D). Instead of annihilating biofilm–liquid interfaces inside of the blisters, in the mutant, neighboring blisters prefer to collapse against each other, which eliminates the high-energy interface between the biofilm and the air. Indeed, during the development of the mutant biofilm, newly emergent blisters move towards, and ultimately join, existing blister groups (Figure 5E; Video 6). The triangular shape of each facet in the Δ*bap1*Δ*rbmC* biofilm is therefore a consequence of the merging of multiple blisters, whose ages and radial lengths decrease from the center to the edge of the aggregate.

**Video 6.** Growth of a *V. cholerae* Δ*bap1*Δ*rbmC* mutant (denoted Δ*BC*) biofilm on medium containing 0.6% agar. Imaging began 5 hr after inoculation and has a total duration of 72 hr with 15 min time steps. The field of view is 24.0 mm × 16.0 mm.

### Mechanical instability and biofilm expansion feed back onto one another

**Figure 6.**
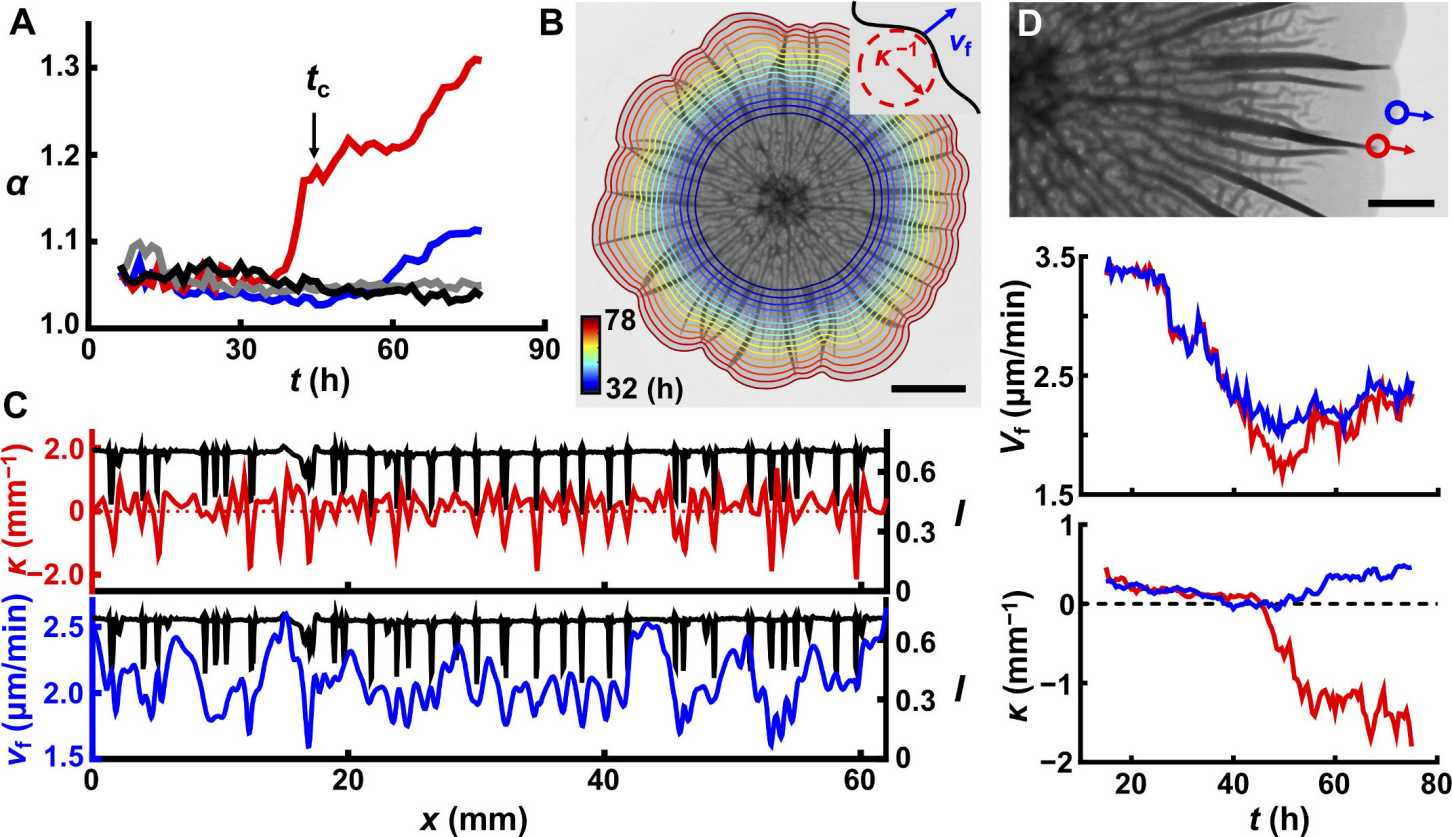
Delamination defines the overall biofilm contour. (**A**) Time evolution of acircularity index *α* (where *α* = *P*^2^/4π*A*, in which *P* is the perimeter of the biofilm and *A* is the area) of the biofilm contour. Two agar substrate concentrations are shown (0.4%, red; 1.0%, blue) for WT *V. cholerae* biofilms. The sharp upturn in *α* defines the critical time *t*_c_. Biofilms lacking matrix (Δ*vpsL* mutant; 0.4%, gray) or possessing an unstructured matrix (Δ*rbmA*Δ*bap1*Δ*rbmC* mutant; 0.4%, black) remain circular. (**B**) Image of a WT *V. cholerae* biofilm grown on 0.7% agar 78 hr after inoculation, overlaid with the time evolution of the biofilm boundary. Colors correspond to the expanding boundary from 32 to 78 hr. Scale bar: 5 mm. *Inset*: schematic of local velocity *V*_f_ and the inverse of local curvature *κ*^−1^. (**C**) Transmitted light intensity profiles *I* (black), *κ* (red), and *V*_f_ (blue) along the biofilm periphery from panel (**B**) at 60 hr. (**D**) *Top*: partial image of the biofilm shown in panel (**B**) at 75 hr. Red and blue dots denote two boundary points at the locations of a delaminated and a flat region, respectively. Arrows indicate boundary expansion. *Middle* and *bottom*: time evolution of *V*_f_ and *κ* of the designated time points during biofilm development. Scale bar: 2 mm. 10.7554/eLife.43920.038Figure 6—source data 1.Local curvature, velocity, and transmission image intensity, and acircularity for biofilm contour evolution dynamics.

We wondered whether the emergence of the 3D biofilm surface topography affected biofilm expansion in the growing plane. One common morphological feature of bacterial biofilms is their irregular petal-shaped 2D contours (Videos 3 and 4). We hypothesized that the evolution of contours could also be a consequence of blister formation. To quantify the contour undulation, we define the acircularity parameter *α* = *P*^2^/4π*A*, in which *P* is the perimeter of the biofilm and *A* is the area (Asally et al., 2012). *α* = 1 for a perfect circle. For a biofilm growing on soft agar (0.4%, Figure 6A), there is a sharp increase in *α* at *t*_c_, the time at which the 3D surface morphology forms at the edge (Figure 6—figure supplement 1). To show that blisters are required for contour undulations, we tracked *α* for mutant biofilms lacking the matrix structural polysaccharide (Δ*vpsL*) (Figure 4—figure supplement 3; Hammer and Bassler, 2003) or lacking matrix structural proteins (Δ*rbmA*Δ*bap1*Δ*rbmC*) (Figure 2—figure supplement 3C; Berk et al., 2012; Yan et al., 2017). In both cases, the biofilm has no surface features and *α* remains close to 1 (Figure 6A).

**Figure 6—figure supplement 1.**
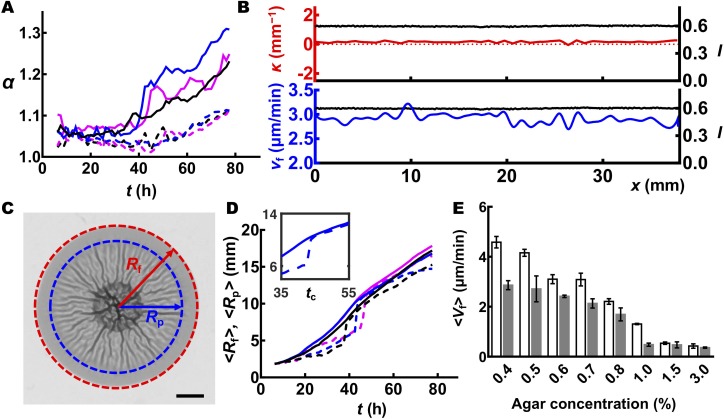
Delamination triggers global and local slowdown of biofilm expansion and shapes the biofilm contour. (**A**) Time evolution of the acircularity index *α* of the biofilm contour. Data are shown in magenta, blue, and black for three biofilms grown on 0.4% (solid curves) and 1.0% (dashed curves) agar plates. (**B**) Profiles of transmitted light intensity *I* (black), local curvature *κ* (red), and local expansion velocity *V*_f_ (blue) along the periphery of the biofilm shown in Figure 6B but at 32 hr. At 32 hr, the wrinkling/delamination pattern had not yet reached the boundary, so the biofilm remained approximately circular, and thus both *I* and *κ* remain constant over the biofilm periphery. *V*_f_ fluctuated modestly, probably because of noise at the growing front and errors in the edge tracking process. (**C**) Image of a WT *V. cholerae* biofilm grown on 0.7% agar taken 30 hr after inoculation. *R*_f_ and *R*_p_ denote the radius of the entire biofilm (outlined in red) and the distal radius to which the morphological pattern extends (outlined in blue), respectively. Scale bar: 2 mm. (**D**) Time evolution of *R*_f_ (solid curves) and *R*_p_ (dashed curves). Data are shown from three different WT *V. cholerae* biofilms (denoted by different colors) grown on 0.4% agar. Initially, *R*_p_ lags behind *R*_f_, indicating a peripheral zone in which the biofilm remains flat and lacks identifiable features. *R*_f_ increases linearly with time, which defines an expansion velocity <*V*_f_> . At a critical time *t’*_c_ (35 hr (black), 42.5 hr (blue), and 45 hr (magenta) for the three cases shown), the region harboring wrinkles or blisters rapidly propagates to the edge of the biofilm (*R*_p_ = *R*_f_). Concurrently, the global biofilm expansion velocity slows, with a crossover time point at *t’*_c_. Inset: close-up view of *R*_f_ and *R*_p_ versus time for one set of data (blue) around *t’*_c_. The critical time that is defined in this manner matches that defined from the *α – t* plot in Figure 6A (i.e. *t*_c_ = *t’*_c_), suggesting that mechanical instability at the biofilm edge triggers the development of contour undulations. (**E**) Averaged biofilm expansion velocity *<V*_f_> before (white) and after (gray) morphological features appear at the rim. *<V*_f_> was calculated by linear fitting of *R*_f_ versus time. Data are represented as mean ± std with *n* = 3. 10.7554/eLife.43920.040Figure 6—figure supplement 1—source data 1.Analysis of contour evolution and biofilm expansion dynamics.

**Figure 6—figure supplement 2.**
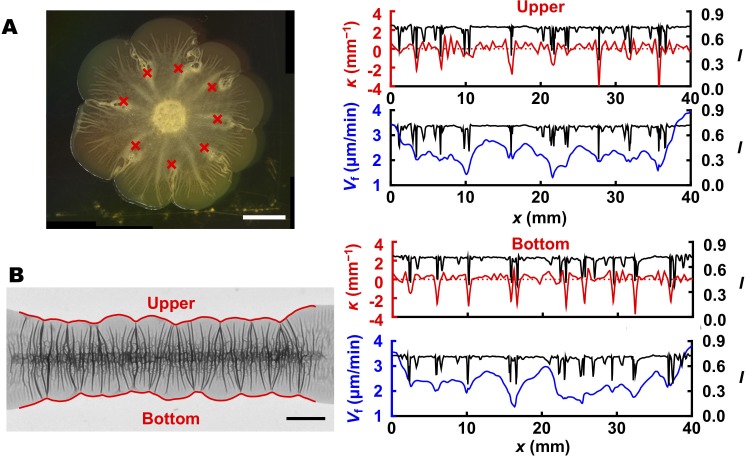
Blister formation drives the overall biofilm contour. (**A**) (*Left*) Bright-field image of a typical 60-hr-old biofilm growing on an agar substrate with predefined defects. The eight surface defects (marked by red crosses) trigger the formation of blisters, which subsequently define the positions of eight ‘petals’ along the biofilm contour. (**B**) (*Left*) Transmission image of a 40-hr-old biofilm grown in a line geometry on an agar substrate. Red outlines indicate the upper and bottom contours. (*Right*) Analyses identical to that provided in Figure 6C were performed for both contours to show that the formation of blisters (indicated by the valleys in the intensity profiles(black)) locally slows down biofilm expansion velocity (blue) and defines the indentation positions along the contour (red). Scale bars: 5 mm. 10.7554/eLife.43920.042Figure 6—figure supplement 2—source data 2.Local curvature, velocity, and transmission image intensity for biofilm contour evolution dynamics in a line geometry.

To investigate the coupling between contour undulations and biofilm morphogenesis in the *z* direction, we followed the time evolution of growing biofilm borders in different geometries (Figure 6B, Figure 6—figure supplement 2). Visually, the indentations along the contours always correspond to the locations of large blisters. To quantify this finding, we measured the local curvature *κ* and expansion velocity *V*_f_ along the biofilm periphery (Figure 6B,C, Figure 6—figure supplements 1 and 2). Both *κ* and *V*_f_ are negatively correlated with the positions of blisters. Monitoring the evolution of a single blister and a nearby flat region shows a transient large difference in *V*_f_ when the blister initially forms at the edge (~ 45 hr in this case; Figure 6D), which triggers the local contour indentation. The emergence of a blister creates an extra dimension into which newly produced biomass can be distributed, which causes local slowing in *V*_f_, thus establishing the correlation between blister locations and negative local curvature. After this transient difference, *V*_f_ becomes comparable for boundaries with and without blisters, and the local curvature reaches a steady value, provided that there is no nearby blister (Figure 4—figure supplement 1). In this steady state, the petal-like contour propagates radially without changing the overall shape of the contour. This explanation for the formation of the biofilm petal shapes suggests that contour undulations require non-homogeneous blister distribution along the biofilm rim and indeed, WT biofilms that are grown on stiff agar (>1.0%) remain nearly circular because they possess regularly and closely spaced blisters (Figure 6A, blue line). As additional evidence for the connection between blister formation and boundary undulation, we show that we can control the number and positions of the petals by specifying the positions of the blisters using patterned substrates (Figure 3, Figure 6—figure supplement 2). We conclude that the 3D surface topography that arises owing to mechanical instabilities caused by biofilm expansion feeds back to slow down expansion and drive contour evolution.

## Discussion

We show here that mechanical instabilities, including wrinkling and delamination, underlie biofilm morphogenesis. Moreover, differences in interfacial energies drive mechanomorphogenesis by dictating the creation or annihilation of new or existing interfaces. Finally, feedback between mechanomorphogenesis and biofilm expansion shapes the overall biofilm contour. Collectively, our findings concerning the connections between a biofilm’s surface morphology and its mechanical and material properties suggest that new genes and/or new compounds that alter biofilm morphology by altering mechanics could be discovered and investigated to address biofilm-related problems.

Morphological patterns can certainly involve gene regulation programs. Nonetheless, we expect our mechanical instability findings in *V. cholerae* biofilms to apply to other systems — from bacteria to humans — because they reveal links between the specific material properties of the biological components and morphological transitions. Regarding bacterial systems, we have already commented on how localized cell death underpins pattern formation at the core of *Bacillus subtilis* biofilms (Asally et al., 2012). In fact, in light of our mechanomorphogenesis model, localized cell death can be viewed as a source of surface defects that functions to trigger delaminations, similar to the defined surface imperfections that drive delaminations shown in Figure 3E. Another example concerns biofilms of *Pseudomonas aeruginosa*, an opportunistic pathogen (Costerton et al., 1999). WT *P. aeruginosa* develops biofilms with a labyrinthine inner pattern surrounded by flat rims (Madsen et al., 2015). By contrast, *P. aeruginosa* mutants that are incapable of phenazine production (Δ*phz*) form biofilm topography similar to those that we examined here for *V. cholerae* with disordered cores surrounded by radial features (Dietrich et al., 2013). We suggest that the mechanical principles uncovered here could also drive the morphological transitions in *P. aeruginosa *biofilms. The WT *P. aeruginosa* biofilm pattern occurs because cells at the biofilm center display upregulated matrix production (Madsen et al., 2015), whereas cells located at the periphery are downregulated for matrix production. In the case of the Δ*phz* mutant, all of the cells overproduce extracellular polysaccharides (Madsen et al., 2015), so we speculate that the Δ*phz P. aeruginosa* mutant forms peripheral radial wrinkles and subsequently delaminations because of the same mechanical instability described here in *V. cholerae*. These examples illustrate how gene regulation and spatially differentiated cell physiology can be coupled to mechanical instability to promote biofilm surface morphologies.

Recent theoretical work on bacterial biofilms has considered mechanical instabilities. Zhang et al. (2016) used simulations to suggest that anisotropic growth coupled with wrinkling instability could generate the surface topography observed in bacterial biofilms, and most recently they considered the possibility of delamination (Zhang et al., 2017). Wang and Zhao (2015) introduced competition between adhesive and elastic energies and computed a phase diagram of the different modes of instability for a film–substrate system. These inspiring theories will be made more valuable by the inclusion of measured biophysical parameters and additional observations generated through experiments. For example, the thin intermediate residual layer that we discovered here is not accurately considered in biofilm simulations, but is required to explain the wrinkling instability in biofilms (Figure 2D). In addition, interfacial energies play a predominant role in driving the morphologies of biological materials that possess soft layers, whereas their roles are minor in classical mechanical systems (Qi et al., 2011). To date, contributions from interfacial energies have been suggested in contexts such as cell sorting in tissues (Brodland, 2002; Foty and Steinberg, 2005), but we are not aware of any work incorporating interfacial energies into mechanical instability models for morphogenesis. Future theoretical analyses can now incorporate measured parameters to understand the rich hierarchical dynamics and the history dependence of mechanomorphogenesis, taking into account biofilm viscoelasticity, interfacial energies, and the consequences of sliding and friction between the biofilm and the substrate (Beroz et al., 2018; Peterson et al., 2015).

Though more sophisticated, eukaryotic organisms often employ similar mechanical instability principles to generate fascinating morphologies. Thus, our findings for biofilms are potentially generalizable and relevant for studies of development in higher organisms (Kim et al., 2015). A close analogy is presented by cerebellum development. The cerebellum possesses a thin, soft layer of Purkinje cells that is sandwiched between the rapidly growing external granular layer and the slow-growing internal granular layer (Lejeune et al., 2016b). Through wrinkling instabilities, the cerebellum develops finely spaced parallel grooves called folia. This hard-soft-hard geometry and the associated wrinkling instabilities directly mirror the configuration that we discovered in *V. cholerae* biofilms. Hence, our work suggests that exploiting mechanical principles to drive key morphogenic events is ancient: it occurs in bacteria, and evolution, as is often the case, has reused prokaryotic processes and principles in eukaryotes. In summary, biofilms represent an intriguing and highly tractable model system to investigate the general role of mechanical forces in morphogenesis, and they provide a convenient system for morpho-engineering.

## Materials and methods

**Key resources table:** 

Reagent type (species) or resource	Designation	Source or reference	Identifiers	Additional information
Strain, strain background (*E. coli*)	S17 λ-*pir*	de Lorenzo and Timmis, 1994		Wild type
Strain, strain background (*V. cholerae*)	C6706*str*2	Thelin and Taylor, 1996		El Tor wild type
Strain, strain background (*V. cholerae*)	JY283	Yan et al., 2017		*vpvC*^W240R^ Δ*pomA* (denoted WT)
Strain, strain background (*V. cholerae*)	JY285	Yan et al., 2017		*vpvC*^W240R^ Δ*pomA*Δ*bap1*Δ*rbmC*
Strain, strain background (*V. cholerae*)	JY286	Yan et al., 2017		*vpvC*^W240R^ Δ*pomA*Δ*rbmA*Δ*bap1*Δ*rbmC*
Strain, strain background (*V. cholerae*)	JY287	Yan et al., 2017		*vpvC*^W240R^ Δ*pomA*Δ*vpsL*
Strain, strain background (*V. cholerae*)	JY370	Yan et al., 2017		*vpvC*^W240R^Δ*pomA lacZ*:P_tac_-*mKate2*:*lacZ*
Strain, strain background (*V. cholerae*)	JY376	Yan et al., 2017		*vpvC*^W240R^Δ*pomA* Δ*vpsL lacZ*:P_tac_-*mKate2*:*lacZ*
Strain, strain background (*V. cholerae*)	JY395	This study		*vpvC*^W240R^Δ*pomA* Δ*bap1*Δ*rbmC lacZ:* P_tac_-*mKate2*:*lacZ*
Recombinant DNA reagent	Plasmid: pKAS32	Skorupski and Taylor, 1996		Suicide vector, Amp^R^ Sm^S^
Recombinant DNA reagent	Plasmid: pNUT144	Drescher et al., 2014		Suicide vector, Amp^R^ Kan^R^ Sm^S^
Recombinant DNA reagent	Plasmid: pNUT157	Drescher et al., 2014		pNUT144 *vpvC*^W240R^
Recombinant DNA reagent	Plasmid: pCMW112	Hammer and Bassler, 2003		pKAS32 Δ*vpsL*
Recombinant DNA reagent	Plasmid: pCN004	Nadell and Bassler, 2011		pKAS32 *lacZ*:P_tac_-*mKate2*:*lacZ*
Recombinant DNA reagent	Plasmid: pCN007	Nadell et al., 2015		pKAS32 Δ*rbmA*
Recombinant DNA reagent	Plasmid: pCN008	Nadell et al., 2015		pKAS32 Δ*rbmC*
Recombinant DNA reagent	Plasmid: pCN009	Yan et al., 2016		pKAS32 Δ*bap1*
Recombinant DNA reagent	Plasmid: pCDN010	Nadell et al., 2015		pKAS32 Δ*pomA*
Software, algorithm	MATLAB and ImageProcessing Toolkit	Mathworks, 2015		https://www.mathworks.com/products/matlab.html
Software, algorithm	PRISM version 6.07	GraphPad, 2015		https://www.graphpad.com/scientific-software/prism/
Software, algorithm	Image composite editor version 2.0.3	Microsoft, 2015		https://www.microsoft.com/en-us/research/project/image-composite-editor/
Software, algorithm	Gmsh version 3.0.6	Geuzaine and Remacle, 2009		https://gmsh.info
Software, algorithm	Paraview version 5.5.0	Ahrens et al., 2005		https://www.paraview.org/
Software, algorithm	FEniCS version 2017.2.0	Alnæs et al., 2015		https://fenicsproject.org/
Software, algorithm	DigiCamControl software version 2.0.72.0	DigiCamControl, 2015		http://digicamcontrol.com/
Software, algorithm	Leica Map Start version 7.4.8051	Leica, 2017		https://www.leica-microsystems.com/products/microscope-software/details/product/leica-map/
Software, algorithm	ImageJ and freehand line selection tool	NIH		https://imagej.nih.gov/ij/
Software, algorithm	RheoPlus version 3.40	Anton Paar, 2008		
Other	LB broth, Miller	ThermoFisher	Cat# BP1426-2	
Other	Bacto agar	VWR	Cat# 214030	
Other	O.C.T. agent	Tissue-Tek, Sakura	Cat# 4583	
Other	Silicone oil, 5 cSt	Sigma Aldrich	Cat# 317667	
Other	Glass beads, acid washed, 425 – 600 µm diameter	Sigma Aldrich	Cat# G8772	
Other	MP Biomedicals Roll & Grow Plating Beads, 4 mm in diameter	ThermoFisher	Cat# MP115000550	
Other	BD PrecisionGlide needles (0.6 mm × 2.5 mm)	Sigma Aldrich	Cat# Z118044	
Other	EMD Millipore,25 mm in diameter	Sigma Aldrich	Cat# VSWP02500	
Other	SytoX Green Nucleic Acid Stain	ThermoFisher	Cat# S7020	
Other	Wheat Germ Agglutinin Sampler Kit	ThermoFisher	Cat# W7024	
Other	Higgins Black India Ink			
Other	Physica MCR 301 shear rheometer	Anton Paar, 2008		
Other	Nikon D3300 SLR digital camera with DX Zoom-Nikkor 18-55 mm lens	Amazon		https://www.amazon.com/Nikon-1532-18-55mm-3-5-5-6G-Focus-S/dp/B00HQ4W1QE/ref=sr_1_3?ie=UTF8&qid=1492108083&sr=8-3&keywords=D3300&th=1
Other	Huion L4S light box	Amazon		https://www.amazon.com/Huion-L4S-Light-Box-Illumination/dp/B00J0UUHPO
Other	Sigma 105 mm macro lens for Nikon DSLR camera	Amazon		https://www.amazon.com/Sigma-258306-105mm-Macro-Camera/dp/B0058NYW3K/ref=sr_1_sc_3?ie=UTF8&qid=1485483491&sr=8-3-spell&keywords=sigma+macroles
Other	Leica stereoscope model M205 FA	Leica		
Other	Leica DCM 3D micro-optical system	Leica		https://www.leica-microsystems.com/products/light-microscopes/upright-microscopes/details/product/leica-dcm-3d/
Other	VR3200 wide-area 3D measurement system	Keyence		https://www.keyence.com/products/measure-sys/3d-measure/vr-3000/models/vr-3200/index.jsp
Other	FEI XL 30 FEG-SEM	FEI		https://iac.princeton.edu/equipment.html
Other	Millrock Technology, BT85A-A	Millrock		https://www.millrocktech.com/
Other	VCR IBS/TM200S ion beam sputterer	VCR		https://iac.princeton.edu/equipment.html

### Bacterial strains

All of the *V. cholerae* strains used in this study are derivatives of *V. cholerae* O1 biovar El Tor strain C6706str2 (Thelin and Taylor, 1996), harboring a missense mutation in the *vpvC* gene (VpvC W240R) (Beyhan and Yildiz, 2007). Bacterial cultures were grown at 37°C under constant shaking in standard lysogeny broth (LB) medium. Genetic engineering of *V. cholerae* was performed using allelic exchange with pKAS32 (Skorupski and Taylor, 1996). All plasmids used in the current study have been reported previously (see 'Key resources table'). pKAS32-derived plasmids were introduced into *V. cholerae* by conjugation with *Escherichia coli* S17 *λ*-*pir* (de Lorenzo and Timmis, 1994), selection on plates containing ampicillin (100 mg/L) and polymyxin B (6 mg/L), and subsequent counterselection on plates containing streptomycin (500 mg/L). Deletions were verified by PCR and phenotypic analysis. The constitutive *mKate2* gene (Shcherbo et al., 2007) is driven by P_tac_ and was inserted into the *V. cholerae* chromosome at the *lacZ* locus (as previously described) with X-Gal (50 mg/L) present in the counterselection step (Nadell and Bassler, 2011).

### Biofilm growth

#### Biofilm growth on agar plates

LB medium solidified with different percentages of agar was used as the solid support to grow biofilms. *V. cholerae* strains were streaked onto LB plates containing 1.5% agar and grown at 37°C overnight. Individual biofilms were selected and inoculated into 3 mL of LB liquid medium containing ~ 10 glass beads (MP Biomedicals Roll and Grow Plating Beads, 4 mm diameter) and the cultures were grown with shaking at 37°C to mid-exponential phase (5–6 hr). Subsequently, the cultures were mixed by vortex to break clusters into individual cells, OD_600_ was measured, and the cultures were back-diluted to an OD_600_ of 0.5. 1 μL of these preparations were spotted onto pre-warmed agar plates. Subsequently, the plates were incubated at 37°C. Typically, four biofilms were grown per agar plate. For time-lapse imaging, one or two biofilms were grown on each plate.

#### Biofilm growth on substrates with defined defects

On prewarmed agar plates, syringe needles (BD PrecisionGlide needles, 0.6 mm × 2.5 mm) were used to punch holes at eight locations, equally separated by 45° around a circle. Marks were made on the bottoms of the Petri dishes to guide our eyes for placement of holes in the agar surface. 1 μL of *V. cholerae* cultures at OD_600_ = 0.5, prepared as described in the preceding paragraph, were spotted at the center of the circle. The diameter of the circle was ~ 1 cm for biofilms grown on 0.6% agar and ~ 0.6 cm for biofilms grown on 1.0% agar. Different circle diameters were used to accommodate the differently sized biofilms that form on soft and stiff agar, and to guarantee that, in both cases, when biofilms expanded to cover the pre-defined defects, they remained flat. Following biofilm growth, the positions of these defects were inferred from the marks drawn on the bottoms of the Petri dishes.

#### Biofilm growth in a line geometry

A *V. cholerae* culture at OD_600_ = 0.5 was prepared as described above. A sterile razor blade was carefully dipped into this culture and dried in air for 1 min. The razor blade was gently touched to the surface of a prewarmed agar plate to initiate biofilm growth.

#### Biofilm growth at the liquid–air interface

First, a biofilm was grown for 24 hr following the procedure describe above. Subsequently, 25 mL of LB medium was gently added from the edge of the agar plate. When the liquid reached the biofilm, the liquid lifted the biofilm off the substrate by capillary force.

### Biofilm imaging

#### Bright-field imaging

Biofilms were imaged with a Leica stereoscope in the reflective (bright field) mode. For biofilms larger than the field of view, multiple overlapping images were acquired manually (3 by 3 or 3 by 2) at different locations in the biofilm. Images from multiple locations in biofilms were stitched together with the Image Composite Editor software from Microsoft to yield the full images of the biofilms while preserving the original resolution. Raw images from the stereoscope contain iridescence as the result of reflections from agar, which were removed by setting the color saturation to zero (i.e. converting to black-and-white images).

#### Transmission imaging

A custom transmission imaging setup was built in a 37°C environmental room to follow biofilm growth. Briefly, an agar plate containing the inoculum was placed on an LED illumination pad (Huion L4S Light Box) and imaged with a Nikon D3300 SLR camera equipped with a Sigma 105 mm F2.8 Macro Lens. The entire setup was covered to exclude light. The camera was controlled using DigiCamControl software. Imaging was started 5 hr after inoculation, at which time the camera was capable of focusing on the growing biofilm. Imaging was performed automatically every 15 min for 3 d. The growth of the biofilm floating at the air–liquid interface was monitored with images acquired at 5 min intervals.

#### Side view imaging

A similar setup to the one described in the preceding paragraph was used to image biofilms from the side, with the following changes. First, the LED illumination pad was placed on the side so that the camera received scattered light from the biofilm surface. Second, an additional camera (Nikon D3300 SLR equipped with DX Zoom-Nikkor 18–55 mm lens) was also placed on the side of the biofilm, at an ~ 90° angle with respect to the first optical path. To remove the optical obstruction from the wall of the agar plate, an imaging window (~ 1 cm × 1 cm) was created using a hot razor blade. Imaging started immediately before the onset of the wrinkling-to-delamination transition, and the time interval between images was 5 min. From time to time, the focus in the side view was adjusted manually.

#### 3D optical profiling

Biofilms were imaged with a Keyence VR-3200 optical profiler using a telecentric multi-triangulation algorithm. Subsequent analyses related to obtaining the 3D profiles of biofilms were performed with the Keyence Analyzer software. In brief, noise was first removed from the raw data using the built-in function in the Keyence Analyzer software to give smooth, continuous surface profiles. Surfaces corresponding to agar were excluded by setting upper and lower height thresholds. 3D views of biofilms were rendered with a built-in function in the software. The corresponding line profiles were extracted along an arc centered at the center of the biofilm.

### Cross-sectioning of biofilms

Biofilms of *V. cholerae* strains expressing *mKate2* were grown on agar plates as described above. Where indicated, 0.5 μM SytoX Green Nucleic Acid Stain (ThermoFisher) was added to the agar to stain dead cells. The region of the agar substrate containing a biofilm (~ 2.5 cm × 2.5 cm) was removed and transferred to an empty petri dish. O.C.T. agent (Tissue-Tek, Sakura) was applied to the surface of the biofilms, and the entire Petri dish was rapidly dipped into a dry ice–ethanol mixture to solidify the O.C.T. agent together with the biofilm. Razor blades were used to cut through the solidified samples. Samples with exposed cross-sections were immediately transferred to a homemade T-shaped sample holder and kept frozen in a dry ice–ethanol mixture. These samples were transferred to a Leica stereoscope and imaged in bright-field mode or in fluorescent mode with an mCherry or GFP filter set.

### Rheological measurements

#### Shear rheology of biofilms

All rheological measurements were performed with a stress-controlled shear rheometer (Anton Paar Physica MCR 301) at 37°C. For each measurement, 100–960 biofilms were collected with a pipette tip or a razor blade and transferred onto the lower plate of the rheometer. After sandwiching the biofilm cells between the upper and lower plates with a gap size of 0.5 mm, silicone oil (5 cSt at 25°C, Sigma Aldrich) was applied to surround the biofilm. Sandblasted surfaces were used for both the upper and lower plates to avoid slippage at the boundary. Oscillatory shear tests were performed with increasing amplitudes of the oscillatory strain *ε’* from 0.01 to 2000% at a fixed frequency of 6.28 rad/s. The storage modulus *G’* was extracted with the RheoPlus software as a function of *ε’*. To extract the plateau shear moduli of biofilms, segmented linear fittings were applied to *G’-ε’* curves on a log-log scale. *G’* varies minimally in the plateau region. We used the fitted* G’* value at *ε’ *= 1% as the modulus of the biofilm *G*_f_. All rheological properties of the biofilm remained roughly constant for at least 48 hr.

#### Shear rheology of agar

LB medium containing different agar concentrations was freshly prepared in 100 mL bottles. The semi-solid medium was heated in a microwave, cooled to ~ 55°C, and added (2 mL) to the lower plate of the rheometer preheated to 60°C. The heated agar solution was subsequently sandwiched between the two rheometer plates with a gap size of 0.5 mm and sealed with silicone oil. The preparation was cooled to 22°C using a cooling rate of 1°C/min. Subsequently, the solid agar was heated to 37°C for measurement. This procedure mimics the sequence of events that agar plates were exposed to during preparation and biofilm growth. Smooth surfaces with TrueGap technology were used. Oscillatory shear tests were performed in the linear elastic region at a fixed frequency of 6.28 rad/s. For data obtained with agar, we averaged 10–20 points in the plateau region of the *G’*(*ε’*) curve to give *G*_s_.

#### Poisson ratio measurement

The Poisson ratio *ν* of the biofilm was estimated by compressing the biofilm in the vertical direction and measuring its bulk modulus. Briefly, a home-built hollow cylinder made of polytetrafluoroethylene with a diameter of 25.5 mm was placed between two parallel plates of a rheometer. The biofilm was loaded into the cylinder to fill its volume. The upper plate of the rheometer (with a diameter of 25 mm) was subsequently lowered with a constant velocity (of between 8 mm/s and 12 mm/s). During this measurement, the shaft does not rotate, but rather acts as a piston to measure the normal force. Using the relationship between normal force and shaft displacement, we calculated the bulk modulus *K* of the biofilm to be ~ 130 kPa; much larger than the shear modulus *G’*. From these data, we could calculate the Poisson’s ratio *ν* = (3*K* – 2*G’*) / 2(3*K + G’*) ≈ 0.495, close to the incompressible limit (*ν* = 0.5).

### Biofilm thickness measurements

The surface profiles of biofilms grown for 48 hr were analyzed with a Leica DCM 3D Micro-optical System. A 10× objective was used to image a 3 mm x 3 mm region covering roughly one quarter of the biofilm, with a *z* step size of 2 μm. To measure the thickness of the residual layer, agar plates containing biofilms were slowly vertically lowered into water to peel the biofilms from the substrate. The entire agar plate was allowed to air dry for 5–10 min to remove liquid remaining from the peeling step. After drying, the above analysis procedure was performed to measure the thickness of the residual layer.

The total thickness of the biofilm *h* and the thickness of the residual layer *h*_r_ were measured using Leica Map software. A three-point flattening procedure was first performed on the agar surface to level the image. Next, line profiles were generated at three different locations spanning the agar surface to the surface of the biofilm or the residual layer. An automatic step-size detection procedure was performed with a built-in function in the software to extract *h* or *h*_r_. The three measured values were averaged to give the value for one biological replicate. The biofilm thickness *h*_f_ was obtained by *h*_f_ = *h − h*_r_.

### SEM sample preparation and imaging

Biofilms were grown on 0.6% agar plates for 2 days as described above. The region of the agar containing a biofilm (~ 2 cm × 2 cm) was separated from the remainder of the agar plate, transferred to a piece of glass, and placed horizontally in a 50 mL conical tube and frozen at −80°C overnight followed by overnight lyophilization (Millrock Technology, BT85A-A). The biofilm samples were sliced with a razor blade to expose blisters, sputter-coated with a 5 nm layer of Pd (VCR IBS/TM200S ion beam sputterer), adhered to an upright SEM stub with conductive tape, and imaged with a scanning electron microscope (FEI XL30 FEG-SEM).

### Characterization of biofilm residual layers

#### Measurement of colony-forming units

 Biofilms grown for 2 days were peeled off of agar substrates using a phosphate-buffered saline solution (PBS) as described previously (Yan et al., 2018). The floating biofilms were collected with clean pipette tips and the corresponding residual layers were removed from the agar using a sterile razor blade. All samples were transferred to 1.5 mL microcentrifuge tubes containing 1 mL PBS and ~ 0.2 mL small glass beads (acid-washed, 425–600 μm diameter, Sigma), vigorously mixed by vortex for 15 min at 37°C to break apart aggregates, serially diluted in PBS, and plated onto LB plates. The LB plates were incubated overnight at 37°C and subsequently assessed for colony forming units (CFU). Four biological replicates were measured, each with two technical replicates. Raw CFU values were normalized by the volume of each biofilm and residual layer, calculated from the radius and thickness of each biofilm and residual layer, respectively.

#### India Ink staining

Biofilms grown for 2 days were peeled off of agar substrates with PBS as described above. 1 mL of Higgins Black India ink solution (10% in PBS) was added to the agar to cover the area containing an intact biofilm or a residual layer, and the preparation was air-dried at room temperature for 30 min. The stained residual layer was subsequently imaged with a Leica stereoscope in the bright-field mode.

### Antibiotic killing assay

Biofilms of *V. cholerae* strains constitutively expressing *mKate2* were inoculated onto semipermeable membranes (EMD Millipore VSWP02500) that had been placed on top of 0.6% agar. The plates were incubated at 37°C for 2 days. The semipermeable membranes were gently removed from the agar surface using tweezers, and subsequently floated at room temperature overnight on top of 3 mL LB medium containing 1.7 μM SytoX Green stain with or without 50 μg/mL tetracycline.

### Biofilm image analyses

Image analyses were performed with custom codes written in MATLAB and with ImageJ software. Raw transmitted light image data were first converted into intensity images. From the pixel intensity distributions, we identified the peak with the highest intensity *I*_b_ and used it as background. We set the minimum intensity *I*_min_ = 0 and the average background intensity *I*_b_ = 0.9 to standardize the contrast of the images. Images were then smoothed with a median filter. From the intensity distribution, we also identified the intensity value *I*_V_ of the valley immediately adjacent to the background peak and used it as the thresholding value to binarize the image (using a built-in thresholding function in MATLAB). We separated the biofilm object *F* from the background. We used the image of each biofilm at *t* = 12 hr after inoculation to define the center *O_F_* for all time points. When mutations affecting biofilm morphology arose, they were manually excluded from the image analysis.

To quantify variations in the amplitudes of biofilm morphological features, we extracted the intensity profiles *I*^E^(*θ*) along a circle near the biofilm edge. We use a built-in function in MATLAB to identify the positions and the prominence Δ*I*_p_ of the peaks in *–I*^E^(*θ*). We set the minimum peak prominence to be 0.02 to eliminate noise.

To extract the periodicity of the wrinkling or delamination pattern, we tracked the time evolution of these patterns from images. For wavelength analysis, we applied fast Fourier transformation (FFT) to intensity functions *I^r^*(*θ*) in a ring at time *t* and radial coordinate *r*, and identified *N*(*r,t*) from the peak frequency in the power spectrum. We also verified the values by autocorrelation and manual counting. We plotted all data from different time points and fitted them with a linear function *N*(*r*)=2π*r*/*λ* to obtain the intrinsic wavelength *λ*. The radial coordinate at which *N* decreases to zero was defined as *R*_p_. For images of biofilms grown in a line geometry, several values of *N* were extracted from multiple lines at different distances from the central line, averaged, and subsequently used to extract *λ*.

For contour analyses, we first obtained the biofilm object *F* from the binarized image. From the binarized object *F*, we extracted the perimeter *P* and the area *A* of region *F.* At each time point, we calculated the acircularity *α* as *α* = *P*^2^*/*4π*A.* To define the radii for biofilms that were not strictly circular, we used <*R*_f_> = <|***r****_i_**–r**_O_*|>*_i_*, averaged over all the points ***r****_i_* on the circumference ∂*F*. <*R*_f_> was then calculated over time to give <*R*_f_(*t*)> versus *t*. Segmented linear regression with two segments was used to quantify the expansion velocity of the biofilm <*V*_f_> before and after the critical time *t*_c_ and to define the critical time itself.

To capture local curvature *κ* and expansion velocity *V*_f_, the smoothed boundary ∂*F* was locally approximated by quadratic polynomials ***r****_i_,*_2_(*t*) at ***r****_i_*. The parametrized curve *x_i_,*_2_(*t*) and *y_i_,*_2_(*t*) allowed us to calculate the analytical curvature $\kappa_{i}$ and normal ***n****_i_* locally using the weighted central difference. Coarse-grained contours at time points *t* and *t*+Δ*t* were then connected by joining ***r****_i_*(*t*) to its nearest neighbor ***r****_i_*(*t*+Δ*t*) in ∂*F_t+Δt_*, yielding local velocities *V*_f*,i*_ = |***r****_i_*(*t*+Δ*t*) – ***r****_i_*(*t*)|/Δ*t*.

To analyze the side-views of blisters, blister contours were manually extracted with ImageJ software and then smoothed. The baseline of the blister was obtained by averaging the *z* coordinate of the left and right bottom region of the blister. The blister height *H* was calculated as the distance between the peak of the blister to the baseline. The width of the blister *W* was measured at half of the blister height.

### Theoretical modeling procedures

We adapted a trilayer model from previous work (Lejeune et al., 2016b), and modeled the biofilm system with the following three elastic components: the biofilm (top), the residual layer (middle), and the agar substrate (bottom) denoted with subscripts f, r, and s, respectively. *V. cholerae* biofilms harbor an active growing top cell layer and a dead cell layer underneath (Figure 4—figure supplement 2). The live and dead cell layers are connected to each other, and they were removed together for our mechanical measurements; so in the model, we do not distinguish between the two and we treat them as a single biofilm layer. Biofilm and residual layers were modeled as thin elastic sheets with thickness *h*_f_ and *h*_r_, whereas the agar substrate was modeled as an elastic body with a thickness *h*_s_, much larger than that of the other two layers. The relevant scale for the continuum model is about the thickness of the film (>50 μm). Therefore, we could neglect potential structural and materials heterogeneities in the biofilm, which exist on a much smaller scale (~ 5 μm, see Yan et al., 2018). The shear modulus and Poisson’s ratio of the materials are denoted by *G* and *ν*, respectively. For theoretical calculations, we treated all three layers as incompressible materials and hence, *ν* = 0.5 (see the above experimental measurements). In the simulation, the residual layer grows at the same rate as the biofilm layer, while the substrate does not grow (as confirmed by comparing the locations of the edge of a biofilm and the residual layer; see Figure 3—figure supplement 1). This growth difference induces a strain mismatch *ε* between the biofilm/residual layer and the substrate.

Following previous studies (Lejeune et al., 2016a), we applied the Föppl-von Kárman equation to the biofilm model. Assuming a sinusoidal profile of the surface undulations, we can write the longitudinal stress *S* in the film as:

$$S\left(n\right)=\;\frac{G_{f}h_{f}^{2}n^{2}}{3}+\frac{\tilde{K}}{h_{f}n^{2}}\;,$$

where n is the wave number and $\tilde{K}$ is the combined stiffness of the residual layer and the substrate layer:

$$\tilde{K}=\;\frac{4G_{s}n}{nh_{r}\left(G_{s}/G_{r}-1\right)+2}\;,$$

and the effective substrate modulus of the composite substrate can be calculated by $G_{s}^{\prime }=\tilde{K}h_{s}^{\prime }$ in which *h*'_s_ is the total depth of the strained region (see Lejeune et al., 2016a for details). By numerically solving the nonlinear equation d*S/*d*n =* 0, we determined the minimal critical value of *S* for mechanical instability and the corresponding *n* gives the critical wavenumber *n*_cr_. The wavelength at the onset of wrinkling was then calculated as $\lambda_{cr}\;=\;2\pi /n_{cr}$. The critical stress and strain were obtained by *S*_cr_ = *S*(*n*_cr_) and $\varepsilon_{cr}$ = *S*_cr_/3*G*_f_, respectively. Theoretical predictions from the bilayer model can simply be calculated by setting *G*_s_ = *G*_r_.

The model described above, despite assuming only small strains, accurately predicted the wavelength and critical stress/strain for finite strains (Lejeune et al., 2016a). We verified that the analytical predictions were in reasonable agreement with results obtained from finite element simulations.

The only unknown parameter in the model is the shear modulus of the residual layer *G*_r_, which is difficult to probe experimentally. Therefore, we treated *G*_r_ as the only fitting parameter. We used *h*_r_/*h*_f_ = 0.3 as an average value from the relevant experimental data and fit the model against the experimental data for wavelength versus stiffness contrast between the biofilm and the agar substrate. Fitting was carried out by minimizing the least-square error between the theoretically predicted and the experimentally measured wavelengths. A bisection method was employed that converged in fewer than 10 iterations.

### Computational modeling procedures

A plane-strain computational model was developed to take into account growth, large deformations, and the nonlinear elasticity of the system. We considered the same planar three-layer structure as above. According to finite strain theory, we define the deformation gradient tensor as *$F_{ij}=\partial x_{i}/\partial X_{j}$*, where *x_i_* and *X_i_* denote the coordinates in the deformed and undeformed configurations, respectively (Ogden, 1997). To incorporate the effect of growth, we further introduced the decomposition of the deformation tensor ***F*** = ***F***_e_***F***_g_ as the product of the growth deformation ***F***_g_ and the elastic deformation ***F***_e_ (Figure 2—figure supplement 4) (Rodriguez et al., 1994). We used $F_{g}=\left(\begin{array}{cc} 1+g & 0\\ 0 & 1 \end{array}\right)$ for the biofilm and residual layers to describe their 1D growth (*g* > 0) in the *X*_1_ direction, and we set ***F***_g_ to be the identity matrix ***I*** for the non-growing agar substrate. The growth-induced compressive strain is thus ε=g/(1+g). To account for the nonlinear stress-strain behavior of materials undergoing large deformations, all three layers were modeled as neo-Hookean materials. The strain energy density of each layer is given by Ogden (1997):

$$\Psi (F_{e})=\frac{\mu_{e}}{2}\left(I_{C}-2-2\ln J\right)+\frac{\lambda_{e}}{2}{\left(\ln J\right)}^{2},$$

where *μ*_e_ and *λ*_e_ are the Lamé parameters, and they are related to the shear modulus *G* and Poisson’s ratio *ν *by

$$\mu_{e}=G,\lambda_{e}=\frac{2G\upsilon }{1-2\upsilon }.$$

*I*_C_ = tr(***F***_e_^T^***F***_e_) is the first invariant of the right Cauchy-Green deformation tensor ***C*** = ***F***_e_^T^***F***_e_, and *J* = det(***F***_e_). The total elastic energy of the system can thus be calculated by

$$\Pi =\int_{\Omega_{f}}^{}\Psi \left(F_{e,f}\right)J_{g,f}dX+\int_{\Omega_{r}}^{}\Psi \left(F_{e,r}\right)J_{g,r}dX+\int_{\Omega_{s}}^{}\Psi \left(F_{e,s}\right)J_{g,s}dX,$$

where *Ω*_f/r/s_ denotes the volume occupied by biofilm/residual/substrate in the initial undeformed reference configuration, and *J*_g_ = det(***F***_g_) specifies the volume element change following growth. We assumed that the present instability pattern always seeks the lowest potential energy among all possible configurations at any time during biofilm growth, neglecting the viscoelasticity and plasticity of the biomaterials that could potentially lead to hysteresis in mechanical instability.

### Finite element simulations

For the computational model, we considered a rectangular domain *Ω = Ω*_f_∪*Ω*_r_∪*Ω*_s_ *=* [0, *L*]×[0, *h*_f_*+h*_r_*+h*_s_] composed of three layers, where *L* denotes the size of the system. We use subscripts 1 and 2 to denote the horizontal and vertical components, respectively. Numerically, the task is to calculate the displacement field *u_i_ = x_i_ - X_i_* that minimizes the total potential energy, that is $u=arg\underset{u\in V_{u}}{\min}\Pi$, where *V**_u_*** is the function space that satisfies the boundary conditions on ***u***. Without loss of generality, we considered a scenario in which the biofilm and residual layers grow together but are confined by the left and right walls of the bottom fixed domain *Ω*, that is, the boundary conditions were set by $u_{1}{|}_{X_{1}=0}=u_{1}{|}_{X_{1}=L}=u_{2}{|}_{X_{2}=0}=0$ (Figure 2—figure supplement 4). The nonlinear constrained minimization problem was implemented in the open-source computing platform FEniCS (Alnæs et al., 2015). The computational model was discretized by first-order triangular elements generated by Gmsh (Geuzaine and Remacle, 2009), and the accuracy of the results was verified by mesh refinements. A growth increment of ∆g = 0.002 was employed in the simulations, up to a maximum of 1. For each step, we computed the equilibrium configuration ***x*** and the Green-Lagrange strain tensor ***e*** = 0.5(***F***_e_^T^***F***_e_ – ***I***) of the system. The critical condition for wrinkling instability was identified as a vertical displacement of the biofilm that surpassed the threshold value (0.01*h*_f_). We further calculated the deviatoric strain tensor *e´_ij_ = e_ij_ –* 0.5*δ_ij_e_kk_* and the von Mises equivalent strain *ε*_vM_ = (2*e´_ij_ e´_ij_* /3)^1/2^ (Jones, 2009) to visualize the strain distribution among the three layers. All results were visualized by Paraview software (Ahrens et al., 2005). For the model parameters, we set *h*_r_/*h*_f_ = 0.3 based on the measured thickness values from experiments, and *h*_s_/*h*_f_ = 10 to represent the thick substrate. The stiffness contrast *G*_r_/*G*_f_ = 0.1 was used according to the optimal fitting value from theoretical curves, and we varied *G*_f_/*G*_s_ from 0.02 to 10 to correspond to the experimental conditions. In all simulations, *L* was set to be larger than 10 times the wavelength to minimize the finite size effect, and the Poisson’s ratios of all three layers were set to be 0.45 to ensure convergence of the algorithm.

### Statistical methods

Error bars correspond to standard deviations of the means. Standard *t*-tests were used to compare treatment groups and are indicated in each figure legend. Tests were always two-tailed and unpaired/paired as demanded by the details of the experimental design. All statistical analyses were performed using GraphPad Prism software.

### Software availability

The custom-written MATLAB scripts and simulation codes used in this study are available at https://github.com/f-chenyi/biofilm-morphogenesis (Fei, 2019; copy archived at https://github.com/elifesciences-publications/biofilm-morphogenesis).

## Data Availability

All data generated or analyzed during this study are included in the manuscript and supporting files. Source data files have been provided for all figures, tables and figure supplements.

## Additional files

Source data 1. Raw data for Supplementary file 1 – Table S1.

Supplementary file 1. Summary of biomaterial parameters for *V. cholerae* biofilms. Table S1 reports the measured biomaterial parameters for *V. cholerae* biofilms grown on different concentrations of agar substrates. These measurements include the shear modulus of the substrate and the biofilm, the thickness of the biofilm and the residual layer, and the wavelength of the biofilm surface pattern.

Transparent reporting form
