# Supplementary material for: Mechanical instability and interfacial energy drive biofilm morphogenesis
Source: eLife. 2019 Mar 8;8:e43920. doi: 10.7554/eLife.43920 (PMC6453567; doi:10.7554/eLife.43920)
Supplement: Supplementary file 1. — Table S1 reports the measured biomaterial parameters for V. cholerae biofilms grown on different concentrations of agar substrates. These measurements include the shear modulus of the substrate and the biofilm, the thickness of the biofilm and the residual layer, and the wavelength of the biofilm surface pattern. [file elife-43920-supp1.docx]

**Table S1: Summary of biomaterial parameters for *V. cholerae* biofilms**

Biomaterial parameters for *V. cholerae* biofilms grown on different concentration agar substrates (denoted Agar conc.). *G*_s_ and *G*_f_ correspond to the shear modulus of the substrate and the biofilm, respectively. *h* and *h*_r_ correspond to the total biofilm thickness and the thickness of the residual layer, respectively. *λ* corresponds to the wavelength of the wrinkles/blisters extracted from time-lapse images.

| **Agar conc.** | ***G*_s_ (kPa)** | ***G*_f_ (kPa)** | ***h* (μm)** | ***h*_r_ (μm)** | ***λ* (μm)** |
| --- | --- | --- | --- | --- | --- |
| 0.4% | 0.14 ± 0.01 | 0.8 ± 0.1 | 57 ± 3 | 2.1 ± 0.9 | 506 ± 11 |
| 0.5% | 0.33 ± 0.04 | 1.1 ± 0.2 | 60 ± 4 | 1.8 ± 0.4 | 445 ± 12 |
| 0.6% | 0.51 ± 0.08 | 1.1 ± 0.1 | 64 ± 6 | 5.8 ± 1.5 | 422 ± 20 |
| 0.7% | 1.4 ± 0.2 | 1.5 ± 0.3 | 68 ± 1 | 7.7 ± 0.7 | 380 ± 20 |
| 0.8% | 1.9 ± 0.2 | 1.2 ± 0.2 | 70 ± 6 | 11 ± 2 | 380 ± 13 |
| 1.0% | 3.8 ± 1.1 | 1.1 ± 0.1 | 96 ± 8 | 30 ± 9 | 334 ± 17 |
| 1.5% | 12 ± 7 | 1.4 ± 0.03 | 118 ± 10 | 48 ± 0.3 | 308 ± 28 |
| 3.0% | 67 ± 10 | 2.4 ± 0.1 | 118 ± 3 | 48 ± 7 | 279 ± 10 |
